# Cytochalasan and Tyrosine-Derived Alkaloids from the Marine Sediment-Derived Fungus ***Westerdykella dispersa*** and Their Bioactivities

**DOI:** 10.1038/s41598-017-12327-1

**Published:** 2017-09-20

**Authors:** Dan Xu, Minghe Luo, Fenglou Liu, Dong Wang, Xuejiao Pang, Ting Zhao, Lulin Xu, Xia Wu, Mingyu Xia, Xiaolong Yang

**Affiliations:** 1 Chongqing Key Laboratory of Natural Product Synthesis and Drug Research, School of Pharmaceutical Sciences, Chongqing University, Chongqing, 401331 P. R. China; 2 School of Life Science and Biopharmaceutics, Shenyang Pharmaceutical University, Shengyang, 110016 P. R. China; 3 School of Traditional Chinese Materia Medica, Shenyang Pharmaceutical University, Shengyang, 110016 P. R. China; 4 School of Agriculture, Ningxia University, Yinchuan, 750021 P. R. China; 5 Department of Pharmacy, Institute of Surgery Research, Daping Hospital, Third Military Medical University, 10 Changjiang Branch Road, Chongqing, 400042 P. R. China

## Abstract

Six new cytochalasans, designated as 18-oxo-19,20-dihydrophomacin C (**1**), 18-oxo-19-methoxy-19,20- dihydrophomacin C (**2**), 18-oxo-19-hydroxyl-19,20-dihydrophomacin C (**3**), 19,20-dihydrophomacin C (**4**), 19-methoxy-19,20-dihydrophomacin C (**5**), 19-hydroxyl-19,20-dihydrophomacin C (**6**), and one new tyrosine-derived alkaloid named as gymnastatin Z (**8**), together with two known compounds, phomacin B (**7**) and triticone D (**9**), were isolated from a solid-substrate fermentation culture of *Westerdykella dispersa* which was derived from marine sediments. Their structures were established on the basis of spectroscopic analysis using 1D and 2D NMR techniques, and comparison of NMR data to those of known compounds. The anti-bacterial and cytotoxic activities assays of all isolated compounds were evaluated against eight human pathogenic bacteria and five human cancer cell lines, respectively. Compound **8** exhibited moderate activity against *B. subtilis* with MIC values of 12.5 *µ*g/mL, while compounds **5**, **7** and **8** displayed moderate inhibitory activities against five human cancer cell lines (MCF-7, HepG2, A549, HT-29 and SGC-7901), with IC_50_ values ranging from 25.6 to 83.7 *µ*M.

## Introduction

The cytochalasans, a diverse group of fungal polyketide synthase-nonribosomal peptide synthetase (PKS-NRPS) hybrid metabolites, have attracted much attention from chemists and pharmacologists in the past nearly 60 years due to their intriguing structures and diverse biological functions^1–4^. This group of metabolites share a perhydroisoindol-1-one skeleton to which connected is a benzyl group (cytochalasins), a *p*-methoxybenzyl group (pyrichalasins), a (indol-3-yl)methyl group (chaetoglobosins), or a 2-methylpropyl group (aspochalasins), and which is fused to a 9- to 15-membered carbocyclic (or oxygen containing) ring at positions C-8 and C-9. In 1966, cytochalasin A and B were first discovered from *Phoma strain* S 298 and *Helminthosporium dematioideum* ^3^, and since then, over 200 related derivatives have been reported from various fungi including ascomycetes as well as basidiomycetes, as exemplified by the genera *Aspergillus*, *Penicillium*, *Chaetomium*, *Zygosporium*, *Phoma*, *Rosellinia*, *Ascochyta*, *Metarhizum*, *Xylaria*, *Phomopsis* or *Hypoxylon* ^4–8^. Various cytochalasans exert a wide range of biological activities, such as interfering with cytokinesis, intracellular motility^9–12^, monosaccharide transport systems^13,14^, or intracellular Ca^2+^ regulation^15^, inhibiting thyroid secretion^16^, or displaying cytotoxic^17,18^, antimicrobial or antiparasitic properties^19–23^.

**Figure 1:**
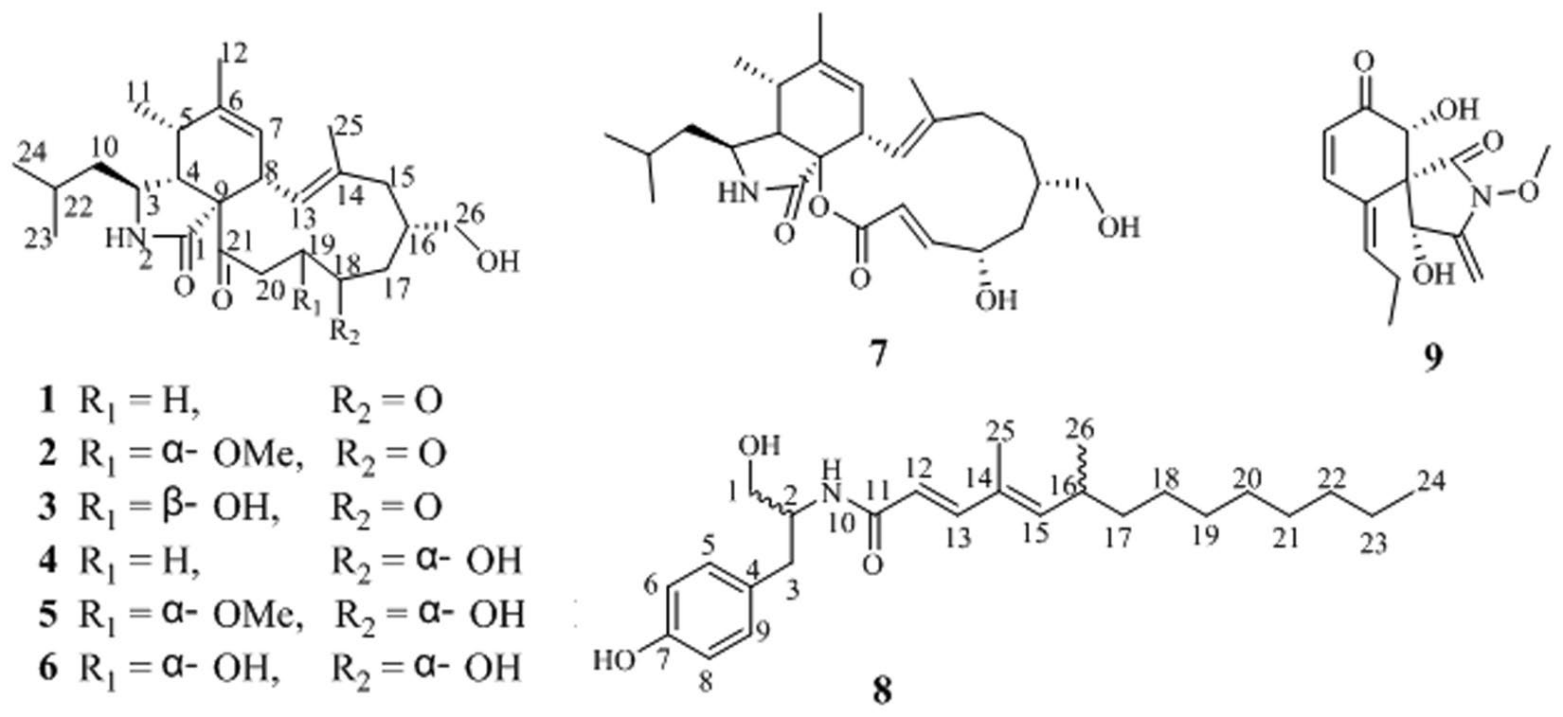
Structures of compounds **1**–**9**.

As part of our program to discover structurally unique and biologically active secondary metabolites from fungi of unique ecological niches, the chemical investigation on *Westerdykella dispersa* was carried out, resulting in the discovery of six new cytochalasans, namely, 18-oxo-19,20-dihydrophomacin C (**1**), 18-oxo-19-methoxy-19,20-dihydrophomacin C (**2**), 18-oxo-19-hydroxyl-19,20-dihydrophomacin C (**3**), 19,20-dihydrophomacin C (**4**), 19-methoxy-19,20-dihydrophomacin C (**5**), 19-hydroxyl-19,20- dihydrophomacin C (**6**), and one new tyrosine-derived alkaloid named as gymnastatin Z (**8**), together with two known compounds, phomacin B (**7**) and triticones D (**9**) (Fig. 1). Herein, we report the fermentation, isolation, structure elucidation, and biological activities of these isolated compounds.

## Results and Discussion

### Structure Elucidation

**Figure 2:**
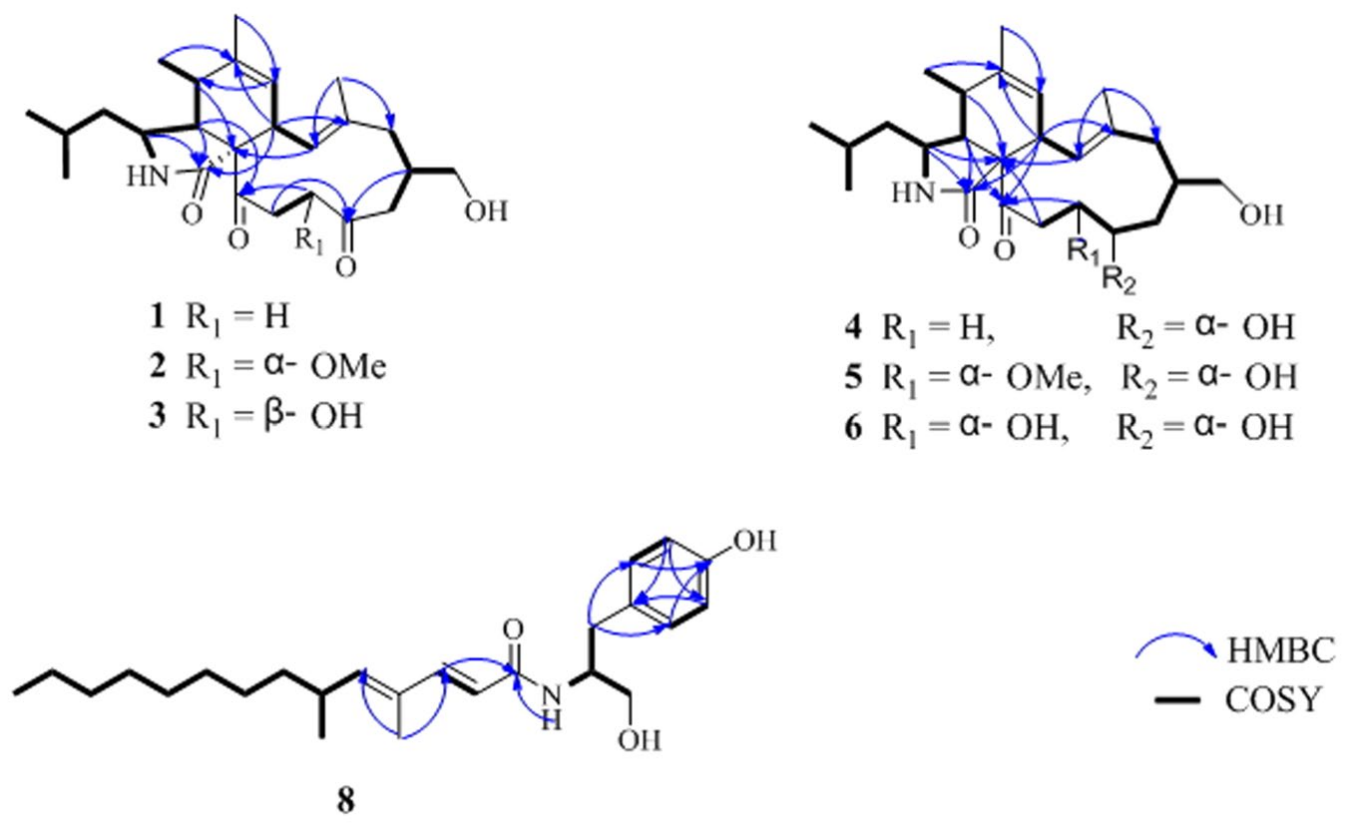
COSY and selected HMBC correlations of compounds **1**–**6** and **8**.

**Figure 3:**
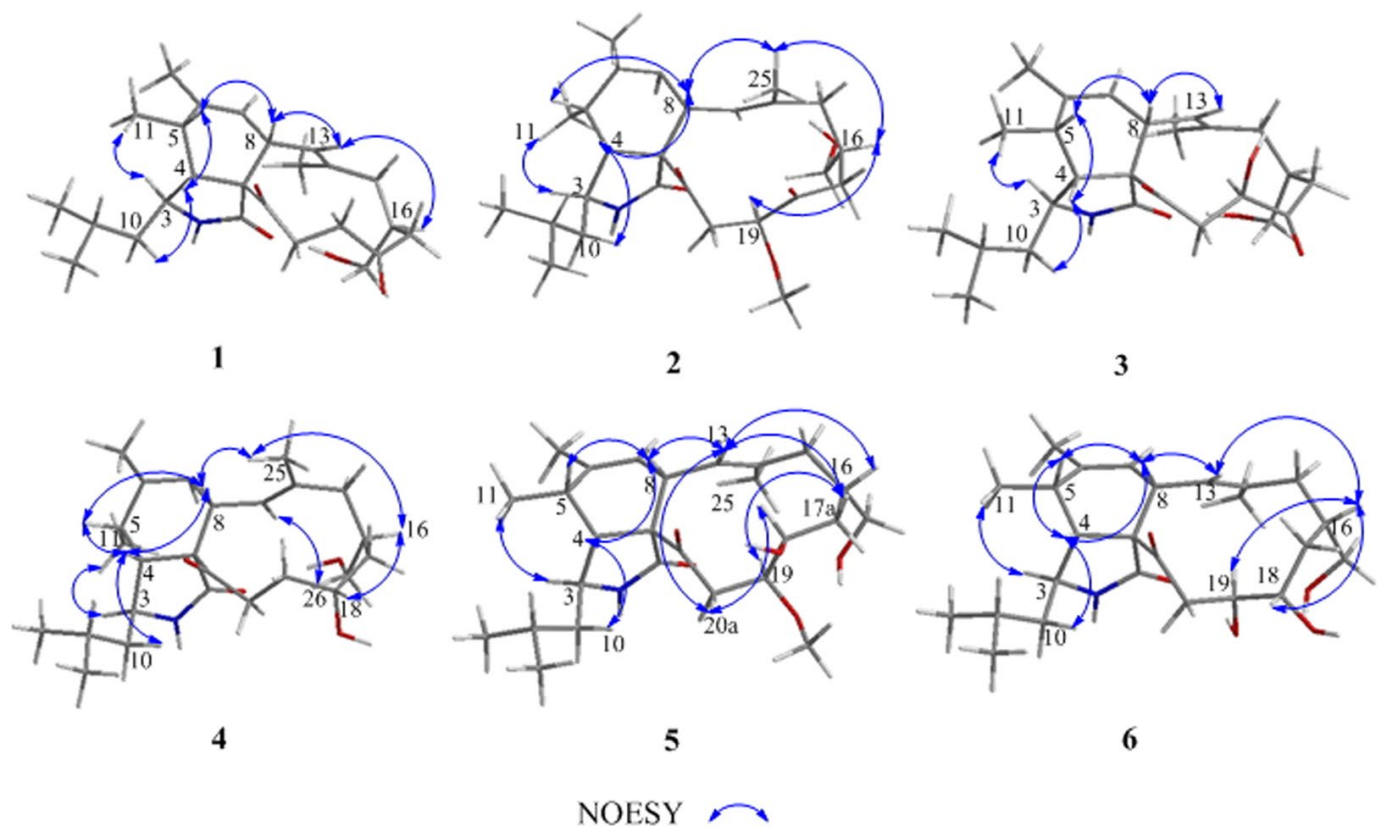
Key NOESY correlations of compounds **1**–**6**.

**Figure 4:**
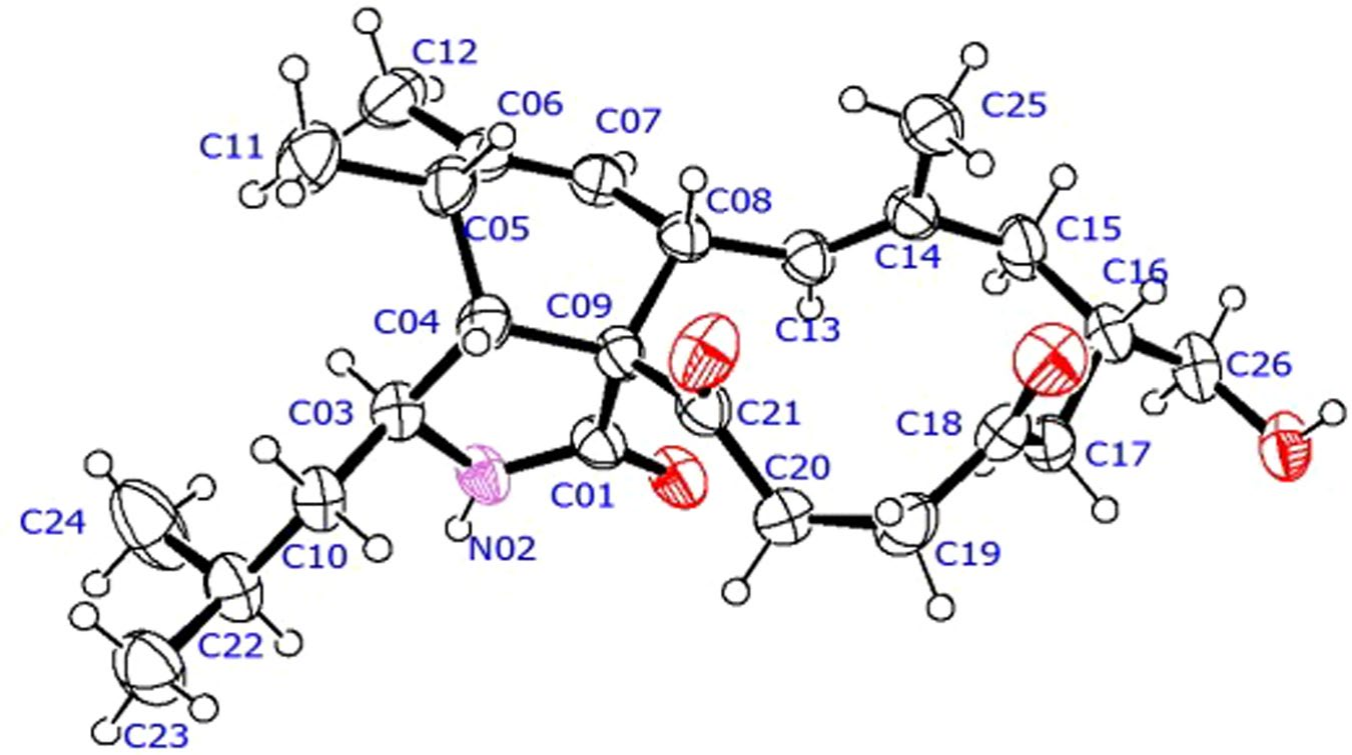
X-ray structure of compound **1**.

Compound **1** was isolated as a colorless block crystal with a molecular formula C_25_H_37_NO_4_, as suggested by the HRESIMS data at *m/z* 438.26152 [M + Na]^+^ (calcd for 438.26148). Interpretation of its^1^H,^13^C NMR, DEPT, and HMQC spectra revealed 25 carbon resonances ascribed to five methyls, six sp^3^ methylenes (one of which oxygenated), six sp^3^ methines, two sp^2^ methines, one sp^3^ nonprotonated carbon, two sp^2^ nonprotonated carbons, and three carboxyl groups. The molecular formula requires eight degrees of unsaturation, but only three carboxyl and four olefinic carbons resonating at *δ*_*C*_ 175.8 (s, C-1), 207.8 (s, C-18), 208.1 (s, C-21), 139.7 (s, C-6), 125.6 (d, C-7), 124.8 (d, C-13), and 136.7 (s, C-14) were detected, indicating the tricyclic nature of **1**. Four spin systems could be detected in the COSY spectrum as depicted in Fig. 2. Detailed analyses of the 1D and 2D NMR spectroscopic data revealed that **1** had a similar structure to phomacin C, a cytochalasan-based alkaloid characterized from *Phoma* sp^2^. The main differences between the two compounds are at positions C-18, C-19 and C-20, with the hydroxyl group (C-18) and the C-19/C-20 trans-olefin in phomacin C being replaced by the two sp^3^ methylenes at positions C-19 and C-20, and ketone substituent at C-18 in **1**. This suggested that the C-19/C-20 *trans*-olefin in phomacin C was reduced, and then oxidative reaction occurred at C-18 to form **1**. The observed HMBC and COSY correlations (Fig. 2) supported the above deduction. On the basis of the above data, the gross structure of **1** was established. The relative configurations of **1** were determined to be 3 S*, 4 R*, 5 S*, 8 S*, 9 S*, and 16 S*, by comparing the NMR data with those reported for phomacin C as well as by the NOESY spectroscopic data (Fig. 3), which were in agreement with those of phomacin C. This was confirmed by the X-ray single-crystal diffraction (Fig. 4) using the anomalous scattering of Mo Kα radiation. It should be noted that the stereochemistry of the cyclohexene and isoindole moieties in all cytochalasans are the same and have been established as 3 S*, 4 R*, 5 S*, 8 S*, 9 S*^1,24^. Therefore, compound **1** was characterized as 18-oxo-19,20-dihydrophomacin C.

**Figure 5:**
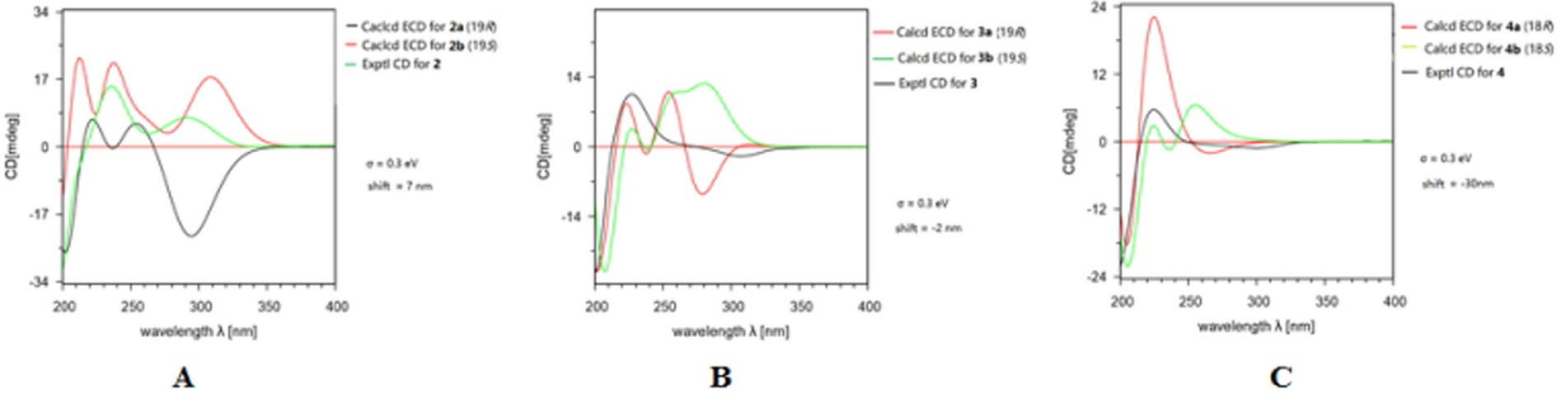
Calculated and experimental ECD spectra of compounds **2**–**4**.

Compound **2** was found to have the molecular formula C_26_H_39_NO_5_ established by HRESIMS at *m/z* 468.27164 ([M + Na]^+^, calcd for 468.27204), suggesting eight degrees of unsaturation. The ^1^H and ^13^C NMR data of **2** (Tables 1 and 2) closely resembled those of **1**, except for the presence of one additional oxygenated methyl and one oxygnated methine, and the absence of one sp^3^ methylene in **2**. This suggested that the methoxylation occurred at C-19 position in **1** to form **2**, evident from HMBC correlations of H-19 with C-21, and H-27 with C-19, combined with correlation of H-19 with H-20 in the COSY spectrum (Fig. 2). The relative configurations of all stereocenters except for C-19 in **2** was characterized the same as in **1** by analysis of NOESY correlations and by comparison of its NMR data with those of **1**. While the absolute configuration of C-19 was determined to be *S* by computational method *via* calculation of the electronic circular dichroism (ECD) (Fig. 5A), which was also supported by the NOESY correlations of H-25 with H-8 and H-16, and H-16 with H-19 indicating H-19 is *β*-oriented (Fig. 3). Therefore, the structure of **2** was characterized as 18-oxo-19-methoxy-19,20-dihydrophomacin C.

**Table 1:** ^1^H NMR data for compounds **1**–**6** in CDCl_3_ (*δ* in ppm, *J* in Hz).

Pos.	1^b^	2^b^	3^a^	4 ^a^	5^a^	6^a^
2	6.66, s	6.07, s	6.17, s	6.24, s	6.60, s	6.20, s
3	3.15, m	3.18, m	3.17, m	3.17, m	3.17, m	3.16, m
4	2.58, m	2.58, m	2.62, m	2.70, m	2.59, m	2.77, dd (2.4, 5.6)
5	2.58, m	2.61, m	2.55, m	2.56, m	2.59, m	2.56, m
7	5.35, s	5.37, brs	5.37, brs	5.44, brs	5.45, brs	5.43, brs
8	3.00, d (10.8)	3.05, d (10.2)	2.97, brd (9.2)	3.08, d (10.8)	3.21, m	3.06, d (10.4)
10a	1.15, m	1.16, m	1.18, m	1.16, m	1.17, m	1.18, m
10b	—	1.30, m	—	—	—	—
11	1.18, d (7.2)	1.21, d (6.6)	1.21, d (6.8)	1.22, d (7.2)	1.21, d (6.6)	1.24, d (7.2)
12	1.72, s	1.75, s	1.74, s	1.76, s	1.76, s	1.77, s
13	6.23, d (10.8)	6.25, d (10.8)	6.23, d (10.2)	6.19, d (10.8)	6.06, d (11.0)	6.19, d (11.2)
15a	2.17, d (12.6)	2.15, d (10.8)	2.16, brd (11.4)	1.98, m	1.88, m	1.95, m
15b	1.80, t (12.0, 24.0)	1.80, t (12.0, 24.6)	1.89, m	1.87, m	1.76, m	1.85, m
16	2.58, m	2.59, m	2.55, m	1.87, m	1.76, m	1.85, m
17a	2.60, m	2.56, m	2.78, m	1.98, m	1.98, m	1.95, m
17b	2.21, d (15.0)	2.30, d (16.0)	2.55, m	1.87, m	1.88, m	1.85, m
18	—	—		3.76, m	3.60, m	3.60, m
19a	2.76, m	5.01, dd (2.4, 10.8)	4.57, brd (8.0)	1.39, m	3.09, m	3.42, m
19b	—	—	—	1.87, m	—	—
20a	3.85, ddd (2.4, 11.4, 17.4)	2.92, m	4.16, dd (11.1, 17.4)	3.60, m	4.11, dd (2.2, 18.7)	4.20, d (17.6)
20b	2.40, ddd (,2.4, 7.8, 17.4)	—	2.55, m	1.92, m	1.93, m	1.90, m
22	1.58, m	1.56, m	1.54, m	1.55, m	1.58, m	1.54, m
23/24	0.90, d (6.6)	0.90, d (6.6)	0.91, d (6.0)	0.89, d (6.8)	0.90, d (6.6)	0.90, d (6.8)
25	1.36, s	1.35, s	1.43, s	1.55, s	1.53, s	1.54, s
26a	3.58, dd (4.8, 10.8)	3.61, dd (5.4, 10.8)	3.66, m	3.60, m	3.55, m	3.56, m
26b	3.41, dd (6.0, 9.6)	3.45, dd (7.2, 10.8)	3.53, t (7.3, 17.0)	3.35, m	3.33 t (9.2, 18.5)	3.34, t (9.2, 18.4)
27	—	3.34, s	—	—	3.51, s	—

^a^Spectra were recorded at 400 MHz. ^b^Spectra were recorded at 600 MHz.

**Table 2:** 13C NMR (100 MHz) data for compounds **1**–**6** in CDCl_3_ (*δ* in ppm).

Pos.	1	2	3	4	5	6
1	175.8, C	174.9, C	175.4, C	175.6, C	175.8, C	174.8, C
3	50.7, CH	50.7, CH	50.7, CH	50.6, CH	50.8, CH	50.6, CH
4	52.5, CH	52.9, CH	51.6, CH	51.3, CH	52.6, CH	50.6, CH
5	35.2, CH	35.3, CH	35.1, CH	35.2, CH	35.5, CH	35.2, CH
6	139.7, C	139.8, C	139.8, C	139.8, C	139.7, C	140.2, C
7	125.6, CH	125.5, CH	125.6, CH	125.7, CH	125.6, CH	125.4, CH
8	43.2, CH	43.9, CH	43.3, CH	43.6, CH	44.1, CH	44.0, CH
9	66.9, C	65.1, C	66.4, C	67.6, C	67.1, C	67.5, C
10	48.6, CH_2_	48.1, CH_2_	48.5, CH_2_	48.5, CH_2_	48.7, CH_2_	48.5, CH_2_
11	13.3, CH_3_	13.3, CH_3_	13.4, CH_3_	13.4, CH_3_	13.4, CH_3_	13.4, CH_3_
12	19.8, CH_3_	19.8, CH_3_	19.8, CH_3_	19.8, CH_3_	19.8, CH_3_	19.8, CH_3_
13	124.8, CH	124.6, CH	123.8, CH	124.6, CH	124.6, CH	124.8, CH
14	136.7, C	137.2, C	137.4, C	135.2, C	134.9, C	135.2, C
15	44.3, CH_2_	44.1, CH_2_	43.9, CH_2_	43.4, CH_2_	43.7, CH_2_	43.9, CH_2_
16	35.1, CH	35.2, CH	33.8, CH	33.0, CH	33.9, CH	33.5, CH
17	42.1, CH_2_	43.2, CH_2_	38.1, CH_2_	35.1, CH_2_	32.6, CH_2_	33.7, CH_2_
18	207.8, C	204.3, C	210.2, C	68.8, CH	72.2, CH	71.3, CH
19	38.0, CH_2_	78.1, CH	74.2, CH	29.2, CH_2_	78.8, CH	70.3, CH
20	37.4, CH_2_	42.5, CH_2_	45.6, CH_2_	35.4, CH_2_	42.9, CH_2_	43.5, CH_2_
21	208.1, C	203.3, C	206.8, C	211.5, C	211.2, C	212.1, C
22	25.0, CH	25.0, CH	25.0, CH	24.9, CH	24.9, CH	25.0, CH
23/24	21.4/23.6, CH_3_	21.5/23.6, CH_3_	21.5/23.5, CH_3_	21.5/23.5, CH_3_	21.5/23.6, CH_3_	21.4/23.5, CH_3_
25	15.3, CH_3_	15.3, CH_3_	15.6, CH_3_	16.1, CH_3_	16.2, CH_3_	16.1, CH_3_
26	67.4, CH_2_	67.3, CH_2_	67.3, CH_2_	68.3, CH_2_	69.1, CH_2_	68.7, CH_2_
27	—	56.9, CH_3_	—	—	58.1, CH_3_	—

Compound **3** had the molecular formula C_25_H_37_NO_5_, as evidenced by the HRESIMS molecular ion at *m/z* 454.25676 ([M + Na]^+^, calcd for 454.25639), requiring eight degrees of unsaturation, which is 14 mass units less than that of **2**. The NMR data (Tables 1 and 2) of **3** revealed nearly identical structural features to those of **2**, except that the methoxy group at C-19 was replaced by a hydroxyl substituent, which was further supported by HMBC and COSY correlations (Fig. 2). This suggested that compound **3** is the non-methylated derivative of **2**. Detailed analyses of its NMR and NOESY data revealed the relative configurations of all stereocenters except for C-19 in **3** are the same as in **2**. Unfortunately, it is difficult to determine the stereochemistry of C-19 through NOESY experiments. Thus, the absolute configuration of C-19 was determined to be *R* through calculation of the electronic circular dichroism (ECD) (Fig. 5B), which is different from that in **2**. Therefore, compound **3** was characterized as 18-oxo-19-hydroxyl- 19,20-dihydrophomacin C.

Compound **4** was obtained as an amorphous white powder. The HRESIMS of **4** displayed a pseudomolecular ion peak at *m/z* 440.27778 [M + Na]^+^ (calcd for C_25_H_39_NO_4_Na, 440.27713), corresponding to the formula of C_25_H_39_NO_4_. The^1^H, and^13^C NMR data were extremely similar to those of **1**, except for the absence of a carboxyl group (*δ*_*C*_ 207.8 (s, C-18)) and the appearance of an additional oxygenated methine (*δ*_*H*_ 3.76 (m, H-18); *δ*_*C*_ 68.8 (d, C-18)) in **4**. This suggested that compound **4** is a reductive derivative of **1**, which was confirmed by the HMBC and COSY experiments (Fig. 2). The relative stereochemistry of all chiral centers except for C-18 were the same as in **1**-**3** and phomacin C based upon coupling constants and chemical shift comparisons, which was further confirmed by the detected NOESY correlations (Fig. 3). While the absolute configuration of C-18 was determined to be *R* through calculation of the electronic circular dichroism (ECD) (Fig. 5C), which is the same as that in phomacin C. Moreover, the optical rotation value of **4** ($[\alpha {]}_{D}^{25}$ −78.8 (*c* 0.118, CHCl_3_)) is also in agreement with that of phomacin C ([α]_D_ −74.6 (*c* 1.0, CHCl_3_)). Thus, compound **4** was identified as 19,20-dihydrophomacin C^24^.

Compound **5**, white amorphous powder, has the molecular formula C_26_H_41_NO_5_, established by HRESIMS at *m/z* 470.28750 [M + Na]^+^ (calcd for 470.28769), implying seven degrees of unsaturation. Interpretation of its^1^H,^13^C NMR, DEPT, and HMQC spectra revealed 26 carbon signals comprising six methyl groups including one oxygenated signal, five methylenes including one oxygenated signal, ten methines including two olefinic and two oxygenated signals, five quaternary carbons including two olefinic signals and two carbonyl groups. Careful analysis of its NMR data revealed features which very closely resembled those of **4**, except for the presence of one additional oxygenated methyl group and one oxygenated methine, and the absence of one sp^3^ methylenes. This suggested that the methoxylation occurred at C-19 in **4** to form **5**, which was further supported by the HMBC correlations of H-19 with C-21, H-27 with C-19, along with the correlations of H-18 with H-19 observed in the COSY spectrum (Fig. 2). The relative stereochemistry of all chiral centers except for C-18 and C-19 were in accord with those of compounds **1**-**4** based upon coupling constants and chemical shift comparisons, which was further confirmed by the NOESY correlations as depicted in Fig. 3. Furthermore, the α-orientations of hydroxyl group at position C-18 and methoxy group at position C-19 were determined by ROESY correlations of H-13 with H-8, H-16, H-17a and H-20a, H-20a with H-18, and H-19 with H-17a, which allowed us to determine the relative configuration of C-18 and C-19 as S* and S*, respectively. Therefore, compound **5** was determined to be 19-methoxy-19,20-dihydrophomacin C.

The molecular formula of **6**, which was obtained as an amorphous white powder, was determined to be C_25_H_39_NO_5_ as deduced by HRESIMS at *m/z* 456.27169 [M + Na]^+^ (calcd for 456.27204), requiring 7 degrees of unsaturation. The molecular weight of **6** was found to be 14 mass units less than that of **5**. Its ^1^H and ^13^C NMR spectra (Tables 1 and 2) showed resonances for five methyls, five methylenes, ten methines, and five quaternary carbons. Comparison of its NMR spectra with compound **5** revealed resonances nearly identical to those found in the spectra of **5**, except that the resonance for OMe-19 were not observed, suggesting that **6** was the non-methylated analogues of **5**. Further analysis of the COSY and HMBC spectra confirmed the structure of **6** as shown in Fig. 1. The relative configurations of **6** are in agreement with those of **5**, by comparison of the ^1^H and ^13^C NMR spectroscopic data with those of **1**, as well as the observed NOESY correlations (Fig. 3). Therefore, compound **6** is identified as 19-hydroxyl-19,20-dihydrophomacin C.

Compound **8**, a colorless viscous oil, was determined to have the molecular formula C_25_H_39_NO_3_ (seven degrees of unsaturation) by its HRESIMS at *m/z* 424.28217 [M + Na]^+^ (calcd for 424.28222). The IR spectrum revealed the presence of hydroxyl (3302 cm^−1^) and carbonyl groups (1650 cm^−1^). Inspection of the ^1^H, ^13^C NMR, DEPT and HSQC data revealed the presence of three methyls, nine methylenes including an oxygenated one, nine methines (seven are sp^2^ carbons), four sp^2^ quaternary carbons including one carboxyl. The presence of a 1,4-disubstituted benzene ring was determined by analysis of the ^1^H and ^13^C NMR spectra [*δ*_*C*_ 129.1 (s, C-4), 130.2 (d, C-5/C-9), 115.6 (d, C-6/C-8), 154.8 (s, C-7); *δ*_*H*_ 7.06 (2 H, d, *J* = 8.0 Hz, H-5/H-9), 6.78 (2 H, d, *J* = 7.6 Hz, H-6/H-8)]. The COSY spectrum as depicted in Fig. 3 revealed an extended spin system comprising H-12 through H-3 to H-26 HMBC, along with the observed HMBC correlations from H-25 to C-13 and C-15, disclosed the presence of a branched aliphatic chain from C-11 to C-24. Detailed analysis of the ^1^H and ^13^C NMR data of **8** revealed the presence of structural features similar to those found in the known compound, gymnastatin H reported from the sponge-derived fungus *Gymnascella dankaliensis*, suggesting compound **8** to be a new gymnastatin derivative^25^. The distinct differences between **8** and gymnastatin H are that the length of the branched aliphatic chain was increased by two methylenes, and the replacing of the carboxylic acid methyl ester group in gymnastatin H by a hydroxymethyl group in **8**, as evident from the COSY correlation of H-1 with H-2, and the HMBC correlation of H-1 with C-3 (Fig. 2). The *E*-forms of all olefinic double bonds of the side chain as same in gymnastatin were deduced on the basis of their respective coupling constants. Unfortunately, the stereochemistry of C-2 and C-16 could not yet be clarified due to scarcity of material. Thus, compound **8** was identified and designated as gymnastatin Z considering that this compound belonged to gymnastatin derivatives and the gymnastatins A–Y have been already reported^26,27^.

Two known compounds **7** and **9** were characterized as phomacin B^24^, and triticone D^28^, respectively, by comparing of their NMR spectroscopic data with those reported in the literature.

### Biological Activity

#### Cytotoxicity Assay

All isolated compounds were evaluated for cytotoxic activity against human breast cancer cells MCF-7, human hepatocellular carcinoma cells HepG2, human lung cancer cells A549, human colon colorectal adenocarcinoma cells HT-29 and human gastric cancer cells SGC-7901 by the MTT method^29^. The results (see Supporting Information) indicated that compounds **5**, **7** and **8** showed moderate activity against all five cell lines, with IC_50_ values ranging from 25.6 to 83.7 *μ*M. In addition, compounds **4** and **6** exhibited moderate inhibitory activity against HT-29 cells, with IC_50_ values 55.5, 49.1 *μ*M, respectively. However, compounds **1**-**3** and **9** displayed no cytotoxicity.

#### Antibacterial Activity

All isolated compounds were evaluated for their antibacterial activity against Gram-positive (*B. subtilis*, *M. luteus*, *B. anthracis* and *S. enterica*) and Gram-negative (*P. vulgaris*, *S. typhimurium*, *E. coli* and *E. aerogenes*) bacteria^30^. The results (see Supporting Information) indicated that only compound **8** exhibited moderate activity against *B. subtilis* with an MIC value of 12.5 *μ*g/mL, and very weak activity against *P. vulgaris*, *S. typhimurium* and *E. coli* with MIC values of 100 *μ*g/mL. None of them are active against *M. luteus* and *S. enterica*.

In conclusion, seven cytochalasan alkaloids including six new ones (**1**–**6**) and one known derivative (**7**), one new tyrosine-derived alkaloid (**8**), and one known 2-pyrrolidinone alkaloid (**9**) were isolated from *Westerdykella dispersa*. To the best of our knowledge, so far only several polyenes including gelastatins A–B and dykellic acid have been reported from the genus *Westerdykella* ^31,32^. Therefore, this is the first report of these types of alkaloids in this genus.

## Materials and Methods

### General Experimental Procedures

Optical rotations were measured on an Autopol I automatic polarimeter (Rudolph). UV spectra were recorded on an Agilent spectrophotometer (Agilent Cary60). IR spectra were run on a Bruker spectrophotometer (TENSOR 27). HRESIMS spectra were performed on a Bruker instrument (FTICRMS, SolariX). Nuclear magnetic resonance (NMR) spectra were recorded on an Agilent DD2 spectrometer (400 MHz and 600 MHz). Crystal data was collected on a SuperNova area detector diffractometer (Agilent Technologies Inc.) Melting point (m.p.) was obtained on SGW X-4A. Silica gel (200–300 mesh, Anhui liangchen Inc, China), and Sephadex LH-20 (Pharmacia Biotech, Uppsala, Sweden) were used for column chromatography (CC). Semi-preparative HPLC separation was carried out on Hanbon newstyle instrument (Hanbon Sci. and tech., Jiangsu, China) equipped with two NP7000 serials pumps (flow rate: 2 mL/min) and an NU3000 serials UV detector using a Hedera C18 column (250mm × 10 mm, 5*μ*m, Hanbon Sci. and tech., Jiangsu, China).

### Fungal Material and Identification

The fungal strain XL602 was isolated from marine sediments, which were collected at South China Sea, Guangzhou, Guangdong province, China, in July 2014. The species was identified to be *Westerdykella dispersa* based on sequence analysis of the ITS region of 18 S rDNA (GenBank Accession No. KY604839), and was deposited at the School of Pharmaceutical Sciences, Chongqing University (Huxi Campus).

### Fermentation, Extraction, and Isolation

The strain was cultured on a plate of potato dextrose agar (PDA) at 28 °C for 7 days. Agar plugs were cut into small pieces (approximately 0.5 × 0.5 × 0.5 cm^3^) under aseptic conditions, and inoculated into four Erlenmeyer flasks (250 mL, 5 pieces per flask) to prepare the seed culture, previously sterilized by autoclaving, each containing 50 mL modified Czapek-Dox medium (glucose 10.0 g, malt sugar 20.0 g, mannitol 20.0 g, corn steep liquor 1.0 g, yeast extract powder 3.0 g, aginomoto 10.0 g, K_2_HPO_4_ 0.5 g, magnesium sulfate 0.3 g, CaCO_3_ 2.0 g, distilled water 1000 mL) and incubated at 25 °C for 2 days on a rotating shaker at 180 rpm/min. The scale-up fermentation was carried out in 8 Erlenmeyer flasks (2 L) (each containing 300 g of rice, 150 mL modified Czapek-Dox medium, 150 mL H_2_O, sterilized for 20 minutes at 121 °C). Every flask was inoculated with 5.0 mL of the spore inoculum and incubated at room temperature for 30 days.

The fungal cultures of *Westerdykella dispersa* were ultrasonically extracted four times with MeOH (each time 4 L). The solvent was removed to give a crude extract (10.8 g). The organic extracts were combined and concentrated under reduced pressure to yield 10.8 g of brown oil. This extract was chromatographed on column chromatography (CC) over SiO_2_ using a stepwise gradient of petroleum ether/acetone gradient system (9:1, 8:2, 8:4, and 5:5) to yield nine fractions, Fr. 1–9. Fr. 4 (0.5 g) was purified by CC over Sephadex LH-20 (CH_2_Cl_2_/MeOH, 1:1), silica gel CC (petroleum ether/acetone, 5:1), and RP-18 (MeOH/H_2_O, 20:80) to afford compound **9** (4.5 mg). Fr. 6 (0.83 g) was subjected to CC over silica gel (petroleum ether/acetone 6:1, 144:1, 1:1) to yield seven subfractions (6a–6 g). Subfraction 6c was separated by repeated CC over Sephadex LH-20 (CH_2_Cl_2_/MeOH, 1:1) and further purified by semi-preparative HPLC using a C18 column (5 *μ*m, 10 × 250 mm, MeOH/H_2_O, 2 mL/min), yielding compounds **1** (17.4 mg, *t*_*R*_ = 21.6 min, 91% MeOH in H_2_O) and **2** (7.3 mg, *t*_*R*_ = 21.9 min, 92% MeOH in H_2_O),. Compounds **5** (24.6 mg, *t*_*R*_ = 8.2 min, 78% MeCN in H_2_O, 3 mL/min) and **8** (3.8 mg, *t*_*R*_ = 22.0 min, 90% MeOH in H_2_O, 2 mL/min) were obtained from subfraction 6d by silica gel CC (CH_2_Cl_2_/MeOH, 25:1) and semi-preparative HPLC (5*μ*m, 10 × 250 mm). Subfraction 6e was purified by Sephadex LH-20 (CH_2_Cl_2_/MeOH, 1:1) and semi-preparative HPLC (5*μ*m, 10 × 250 mm, MeOH/H_2_O, 2 mL/min) to afford compound **4** (8.1 mg, *t*_*R*_ = 36.2 min, 75% MeOH in H_2_O). Subfraction 6 f was fractionated on Sephadex LH-20 (CH_2_Cl_2_/MeOH, 1:1), semi-preparative HPLC (5*μ*m, 10 × 250 mm, MeOH/H_2_O, 2 mL/min), and silica gel (petroleum ether/EtOAc, 1:1.5–1:2) to yield compound **3** (8.1 mg, *t*_*R*_ = 36.0 min, 70% MeOH in H_2_O) and **7** (2.1 mg). Compound **6** (7.5 mg, *t*_*R*_ = 26.0 min, 75% MeOH in H_2_O) was obtained from Fr. 7 (0.9 g) by CC over Sephadex LH-20 (MeOH/H_2_O, 1:1), repeatedly silica gel (CHCl_3_/MeOH, 50:1–1:1), and semi-preparative HPLC (5*μ*m, 10 × 250 mm, MeOH/H_2_O, 2 mL/min).

1*8-Oxo-*1*9*,2*0-dihydrophomacin C (* ***1*** *):* colorless block crystal; $[\alpha {]}_{D}^{26}$ –79.8° (*c* 0.104, CHCl_3_); UV (MeOH) *λ*_max_ (log *ε*) 202 (4.4) nm; (KBr) *ν*_max_ 3508, 3341, 3027, 2960, 2931, 1712, 1690, 1462 cm^−1^; ^1^H and ^13^C NMR data see Tables 1 and 2; HRESIMS *m*/*z* 438.26152 [M + Na]^+^ (calcd for C_25_H_37_NO_4_Na, 438.26148). Melting point, 136.3–137.1 °C. X-ray crystallographic data of **6**: C_25_H_37_NO_4_, monoclinic, space group: *P*21, *a* = 9.3417 (2) Å, *b* = 10.9357 (3) Å, *c* = 24.5671 (6) Å, *α* = 90°, *β* = 91.595 (2)°, *γ* = 90°, *V* = 2508.75(11) Å^3^, *Z* = 4, D_calcd_ = 1.100 g/cm^3^, *R*_*1*_(*I* > 2*σ*(*I*)) = 0.0562, *wR*_*2*_ = 0.1332. Crystal size, 0.32 × 0.29 × 0.25 mm^3^. Flack parameter. = 0.1(4).

*18-Oxo-19-methoxy-19,20-dihydrophomacin C (* ***2*** *):* white amorphous powder; $[\alpha {]}_{D}^{25}$ –29.4 (*c* 0.143, CHCl_3_); UV (MeOH) *λ*_max_ (log *ε*) 205 (3.3) nm; (KBr) *ν*_max_ 3197, 1719, 1688, 1458 cm^−1^; ^1^H and ^13^C NMR data see Tables 1 and 2; HRESIMS *m*/*z* 468.27164 [M + Na]^+^ (calcd for C_26_H_39_NO_5_Na, 468.27204).

*18-Oxo-19-hydroxyl-19,20-dihydrophomacin C (* ***3*** *):* white amorphous powder; $[\alpha {]}_{D}^{21}$ –42.6 (*c* 0.108, CHCl_3_); UV (MeOH) *λ*_max_ (log *ε*) 208 (3.05) nm; (KBr) *ν*_max_ 3323, 1689, 1507, 1458 cm^−1^; ^1^H and ^13^C NMR data see Tables 1 and 2; HRESIMS *m*/*z* 454.25676 [M + Na]^+^ (calcd for C_25_H_37_NO_5_Na, 454.25639).

*19,20-Dihydrophomacin C (* ***4*** *):* white amorphous powder; $[\alpha {]}_{D}^{25}$ –78.8 (*c* 0.118, CHCl_3_); UV (MeOH) *λ*_max_ (log *ε*) 204 (4.7) nm; (KBr) *ν*_max_ 3345, 3212, 2958, 2927, 2871, 1689, 1453, 1385, 1036 cm^−1^; ^1^H and ^13^C NMR data see Tables 1 and 2; HRESIMS *m*/*z* 440.27778 [M + Na]^+^ (calcd for C_25_H_39_NO_4_Na, 440.27713).

*19-Methoxy-19,20-dihydrophomacin C (* ***5*** *):* white amorphous powder; $[\alpha {]}_{D}^{25}$ –114.4 (*c* 0.104, CHCl_3_); UV (MeOH) *λ*_max_ (log *ε*) 205 (4.9) nm; IR (KBr) *ν*_*max*_ 3197, 2925, 1691, 1453, 1378, 1262, 1098, 1033, 803, 758, 668, 533 cm^−1^; ^1^H and ^13^C NMR data see Tables 1 and 2; HRESIMS *m*/*z* 470.28750 [M + Na]^+^ (calcd for C_26_H_41_NO_5_Na, 470.28769).

*19-Hydroxyl-19,20-dihydrophomacin C (* ***6*** *):* white amorphous powder; $[\alpha {]}_{D}^{26}$ –61.4 (*c* 0.101, CHCl_3_); UV (MeOH) *λ*_max_ (log *ε*) 202 (5.1) nm; IR (KBr) *ν*_max_ 3428, 2927, 2361, 1690, 1452, 1385, 1059 cm^−1^; ^1^H and ^13^C NMR data see Tables 1 and 2; HRESIMS *m*/*z* 456.27169 [M + Na]^+^ (calcd for C_25_H_39_NO_5_Na, 456.27204).

*Gymnastatin Z (* ***8*** *):* colorless viscous oil; $[\alpha {]}_{D}^{22}$ –55.6 (*c* 0.054, EtOH); UV (EtOH) *λ*_max_ (log *ε*) 265 (6.5) nm; IR (KBr) *ν*_max_ 3302, 2957, 2925, 2854, 1650, 1612, 1544, 1516, 1456 cm^−1^; ^1^H and ^13^C NMR data see Table 3; HRESIMS *m*/*z* 424.28217 [M + Na]^+^ (calcd for C_25_H_39_NO_3_Na, 424.28222).

**Table 3:** ^1^H NMR and ^13^C NMR for compound **8** and gymnastatin H in CDCl_3_ (*δ* in ppm, *J* in Hz).

Pos.	8^a^		gymnastatin H^b^	
	*δ*_*H*_	*δ*_*C*_	*δ*_*H*_	*δ*_*C*_
1a	3.71, m	64.4, CH_2_		172.3, C
1b	3.61, dd (5.2, 10.8)			
2	4.22, m	53.3, CH	4.97, dt (7.8, 5.8)	53.3, CH
3	2.82, d (6.0)	36.2, CH_2_	a 3.06, dd (14.1, 5.8)	37.3, CH_2_
			b 3.13, dd (14.1, 5.8)	
4	—	129.1, C	—	127.8, C
5/9	7.06, d (8.0)	130.2, CH	6.97, d (8.5)	130.5, CH
6/8	6.78, d (7.6)	115.6, CH	6.74, d (8.5)	115.5, CH
7	—	154.8, C	—	154.8, C
10	5.86, d (7.6)	—	5.94, d (7.8)	—
11	—	167.6, C	—	166.2, C
12	5.71, d (15.2)	117.2, CH	5.73, d (15.3)	117.1, CH
13	7.23, d (15.2)	147.1, CH	7.24, d (15.3)	147.2, CH
14	—	130.8, C	—	130.9, C
15	5.64, d (9.6)	148.2, CH	5.64, d (9.8)	148.2, CH
16	2.49, m	33.2, CH	2.51, m	33.2, CH
17a	1.27, m	37.2, CH_2_	1.26, m	37.3, CH_2_
17b	1.33, m		1.33, m	
18	1.24, m	27.5, CH_2_	1.22, m	27.5, CH_2_
19	1.24, m	29.3, CH_2_	1.22, m	29.4, CH_2_
20	1.24, m	29.7, CH_2_	1.23, m	31.8, CH_2_
21	1.24, m	29.6, CH_2_	1.25, m	22.7, CH_2_
22	1.24, m	31.9, CH_2_	0.88, t (6.7)	14.1, CH_3_
23	1.33, m	22.7, CH_2_	1.75, s	12.5, CH_3_
24	0.88, t (6.4, 13.6)	14.1, CH_3_	0.97, d (6.6)	20.6, CH_3_
25	1.74, s	12.5, CH_3_	—	—
26	0.97, d (6.8)	20.5, CH_3_	—	—

^a^Spectra were recorded at 400 MHz for ^1^H and at 100 MHz for ^13^C in CDCl_3_.

^b^Spectra were recorded at 300 MHz ^1^H and at 75 MHz for ^13^C in CDCl_3_.

### Cytotoxicity Assay

Cytotoxicity activity was evaluated against MCF-7, HepG2, A549, HT-29 and SGC-7901 by the MTT method^29^. All cell lines was grown in RPMI-1640 medium (GIBCO) supplemented with 10% heat-inactivated bovine serum, 2 nM glutamine, 10^5^ IU/L penicillin, 100 mg/L streptomycin and 10 mM HEPES, pH 7.4. Cells were kept at 37 °C in a humidified 5% CO_2_ incubator. An aliquot (180 *μ*L) of these cell suspensions at a density of 1500 cells mL^−1^ was pipetted into 96-well microtiter plates. Subsequently, 180 *μ*L of sample (in DMSO) at different concentrations was added to each well and incubated for 72 h at the above conditions in a CO_2_ incubator. MTT solution (20 *µ*L of 5 mg/L in RPMI-1640 medium) was added to each well and further incubated for 4 h at 37 °C. After addition of 100 *µ*L DMSO and incubation for 1 h, the cells were lysed to liberate the formed formazan crystals. The optical density (OD) was read on a Multiscan plate reader at a wavelength of 492 nm. DMSO control well, in which sample was absent, was included in the experiment in order to eliminate the influence of DMSO. The inhibitory rate of cell proliferation was calculated by the following formula:

1 $${\rm{Growth}}\,{\rm{inhibition}}( \% )=[1-({{\rm{OD}}}_{{\rm{treated}}}-{{\rm{OD}}}_{{\rm{blank}}})/({{\rm{OD}}}_{{\rm{control}}}-{{\rm{OD}}}_{{\rm{blank}}})]\times 100 \% $$

The cytotoxicity of samples on tumor cells was expressed as IC_50_ values and calculated by LOGIT method.

### Antibacterial Assay

All isolated compounds were evaluated for their antibacterial activity against Gram-positive (*B. subtilis, M. luteus, B. anthracis* and *S. enterica*) and Gram-negative (*P. vulgaris, S. typhimurium, E. coli* and *E. aerogenes*) bacteria. They were grown in liquid LB medium (yeast extract 5 g/L, peptone 10 g/L, NaCl 10 g/L, pH = 7.4) overnight at 37 °C, and the diluted bacterial suspension (10^6^ CFU per milliliter) was ready for detection. The minimum inhibitory concentrations (MIC) of samples and positive control were determined in sterile 96-well plates by the modified broth dilution test^30^. All of wells were filled with 180 *μ*L of bacterial suspension containing 10^6^ CFU per milliliter. Test samples (20 *μ*L) with their different concentrations were added into each well. Medium containing DMSO was used as a negative control, ciprofloxacin was used as the positive control. The final concentrations of ciprofloxacin and test compounds were 100, 50, 25, 12.5, 6.25, 3.125, 1.5625, 0.78125 *μ*g/mL in medium. After incubation, the minimum inhibitory concentration (MIC) was defined as the lowest test concentration that completely inhibited the growth of the test organisms.

## Electronic supplementary material

Supporting information
