# Supplementary material for: Cytochalasan and Tyrosine-Derived Alkaloids from the Marine Sediment-Derived Fungus Westerdykella dispersa and Their Bioactivities
Source: Sci Rep. 2017 Sep 20;7:11956. doi: 10.1038/s41598-017-12327-1 (PMC5607321; doi:10.1038/s41598-017-12327-1)
Supplement: Supplementary file 1 — Supporting information [file 41598_2017_12327_MOESM1_ESM.doc]

Cytochalasan and Tyrosine-Derived Alkaloids from the Marine Sediment-Derived Fungus *Westerdykella dispersa* and Their Bioactivities

Dan Xu^1^, Minghe Luo^5^, Fenglou Liu^4^, Dong Wang^3^, Xuejiao Pang^1^, Ting Zhao^1^, Lulin Xu^1^, Xia Wu^1^, Mingyu Xia,^2^,*, Xiaolong Yang^1^,*

^1^ Chongqing Key Laboratory of Natural Product Synthesis and Drug Research, School of Pharmaceutical Sciences, Chongqing University, Chongqing 401331, P. R. China;

^2^ School of Life Science and Biopharmaceutics, Shenyang Pharmaceutical University, Shengyang 110016, P. R. China;

^3^ School of Traditional Chinese Materia Medica, Shenyang Pharmaceutical University, Shengyang 110016, P. R. China;

^4^ School of Agriculture, Ningxia University, Yinchuan 750021, P. R. China;

^5^ Department of Pharmacy, Institute of Surgery Research, Daping Hospital, Third Military Medical University, 10 Changjiang Branch Road, Chongqing 400042, P. R. China;

***** Correspondence: yxl19830915@163.com; Tel.: +86-23-65678450

**Contents page**

**Figure S1.** ^1^H NMR spectrum of **1** (CDCl_3_, 400MHz)..................................................5

**Figure S2.** ^13^C NMR spectrum of **1** (CDCl_3_, 100 MHz)...............................................5

**Figure S3.** COSY spectrum of **1** (CDCl_3_)...... ...............................................................6

**Figure S4.** HMQC spectrum of **1** (CDCl_3_)....................................................................6

**Figure S5.** HMBC spectrum of **1** (CDCl_3_)....................................................................7

**Figure S6.** NOESY spectrum of **1** (CDCl_3_)...................................................................7

**Figure S7.** HRESIMS spectrum of **1**.............................................................................8

**Figure S8.** IR spectrum of **1**..........................................................................................8

**Figure S9.** UV spectrum of **1** (CH_3_OH)........................................................................8

**Figure S10.** ^1^H NMR spectrum of **2** (CDCl_3_, 400 MHz)..............................................9

**Figure S11.** ^13^C NMR spectrum of **2** (CDCl_3_, 100 MHz).............................................9

**Figure S12.** COSY spectrum of **2** (CDCl_3_).................................................................10

**Figure S13.** HMQC spectrum of **2** (CDCl_3_)................................................................10

**Figure S14.** HMBC spectrum of **2** (CDCl_3_)................................................................11

**Figure S15.** NOESY spectrum of **2** (CDCl_3_)...............................................................11

**Figure S16.** HRESIMS spectrum of **2**.........................................................................12

**Figure S17.** IR spectrum of **2**......................................................................................12

**Figure S18.** UV spectrum of **2** (CH_3_OH)....................................................................12

**Figure S19.** ^1^H NMR spectrum of **3** (CDCl_3_, 400 MHz)............................................13

**Figure S20.** ^13^C NMR spectrum of **3** (CDCl_3_, 100 MHz)...........................................13

**Figure S21.** COSY spectrum of **3** (CDCl_3_).................................................................14

**Figure S22.** HMQC spectrum of **3** (CDCl_3_)................................................................14

**Figure S23.** HMBC spectrum of **3** (CDCl_3_)................................................................15

**Figure S24.** NOESY spectrum of **3** (CDCl_3_)...............................................................15

**Figure S25.** HRESIMS spectrum of **3**.........................................................................16

**Figure S26.** IR spectrum of **3**......................................................................................16

**Figure S27.** UV spectrum of **3** (CH_3_OH)....................................................................16

**Figure S28.** ^1^H NMR spectrum of **4** (CDCl_3_, 600 MHz)............................................17

**Figure S29.** ^13^C NMR spectrum of **4** (CDCl_3_, 100 MHz)...........................................17

**Figure S30.** COSY spectrum of **4** (CDCl_3_)..................................................................18

**Figure S31.** HMQC spectrum of **4** (CDCl_3_)................................................................18

**Figure S32.** HMBC spectrum of **4** (CDCl_3_)................................................................19

**Figure S33.** NOESY spectrum of **4** (CDCl_3_)...............................................................19

**Figure S34.** HRESIMS spectrum of **4**.........................................................................20

**Figure S35.** IR spectrum of **4**......................................................................................20

**Figure S36.** UV spectrum of **4** (CH_3_OH)....................................................................20

**Figure S37.** ^1^H NMR spectrum of **5** (CDCl_3_, 400MHz)..............................................21

**Figure S38.** ^13^C NMR spectrum of **5** (CDCl_3_, 100 MHz)...........................................21

**Figure S39.** COSY spectrum of **5** (CDCl_3_).................................................................22

**Figure S40.** HMQC spectrum of **5** (CDCl_3_)................................................................22

**Figure S41.** HMBC spectrum of **5** (CDCl_3_)................................................................23

**Figure S42.** NOESY spectrum of **5** (CDCl_3_)...............................................................23

**Figure S43.** HRESIMS spectrum of **5**.........................................................................24

**Figure S44.** IR spectrum of **5**......................................................................................24

**Figure S45.** UV spectrum of **5**.....................................................................................24

**Figure S46.** ^1^H NMR spectrum of **6** (CDCl_3_, 600 MHz).............................................25

**Figure S47.** ^13^C NMR spectrum of **6** (CDCl_3_, 100 MHz)...........................................25

**Figure S48.** COSY spectrum of **6** (CDCl_3_)..................................................................26

**Figure S49.** HMQC spectrum of **6** (CDCl_3_)................................................................26

**Figure S50.** HMBC spectrum of **6** (CDCl_3_)................................................................27

**Figure S51.** NOESY spectrum of **6** (CDCl_3_)...............................................................27

**Figure S52.** HRESIMS spectrum of **6**.........................................................................28

**Figure S53.** IR spectrum of **6**......................................................................................28

**Figure S54.** UV spectrum of **6** (CH_3_OH)....................................................................28

**Figure S55.** ^1^H NMR spectrum of **8** (CDCl_3_, 400 MHz).............................................29

**Figure S56.** ^13^C NMR spectrum of **8** (CDCl_3_, 100 MHz)...........................................29

**Figure S57.** COSY spectrum of **8** (CDCl_3_)..................................................................30

**Figure S58.** HMQC spectrum of **8** (CDCl_3_)................................................................30

**Figure S59.** HMBC spectrum of **8** (CDCl_3_)................................................................31

**Figure S60.** HRESIMS spectrum of **8**.........................................................................32

**Figure S61.** IR spectrum of **8**......................................................................................32

**Figure S62.** UV spectrum of **8** (EtOH).......................................................................32

**Table 4.** Antibacterial Activity of Compounds **1**–**9** (MIC, *μ*g/mL).............................33

**Table 5.** Cytotoxic Activities of Compounds **1**–**9** (IC_50_, *μ*M)......................................34

**Table 6.** X-ray crystallographic data of compound **1**...................................................34

**S1.** Computation Section.............................................................................................35

**Figure S1.** ^1^H NMR spectrum of **1** (CDCl_3_, 600MHz)


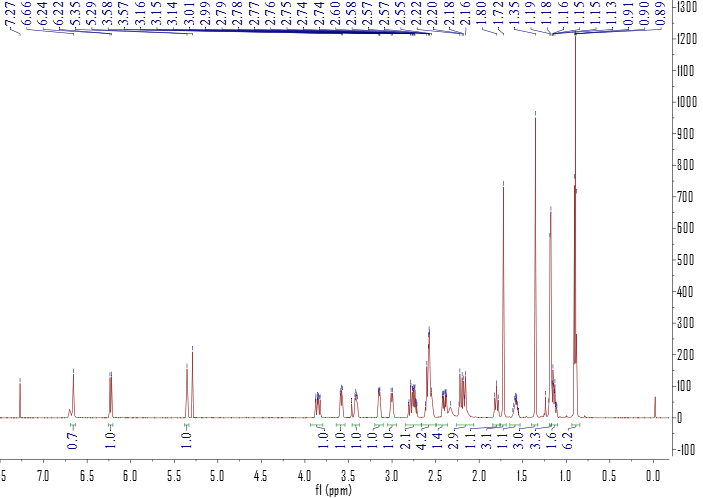


**Figure S2.** ^13^C NMR spectrum of **1** (CDCl_3_, 100 MHz)


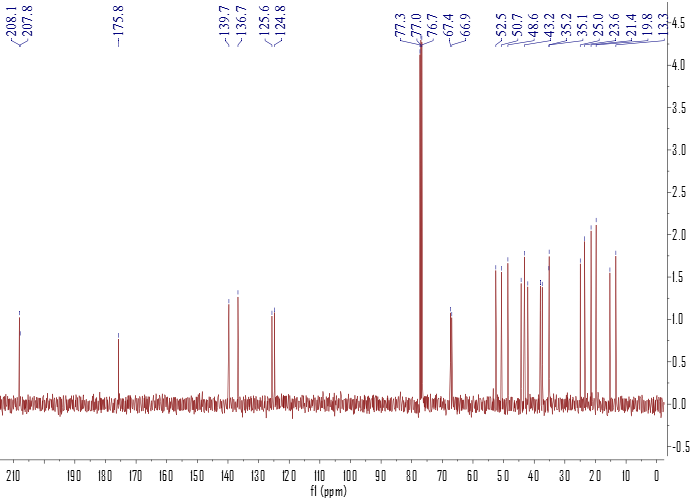


**Figure S3.** COSY spectrum of **1** (CDCl_3_)


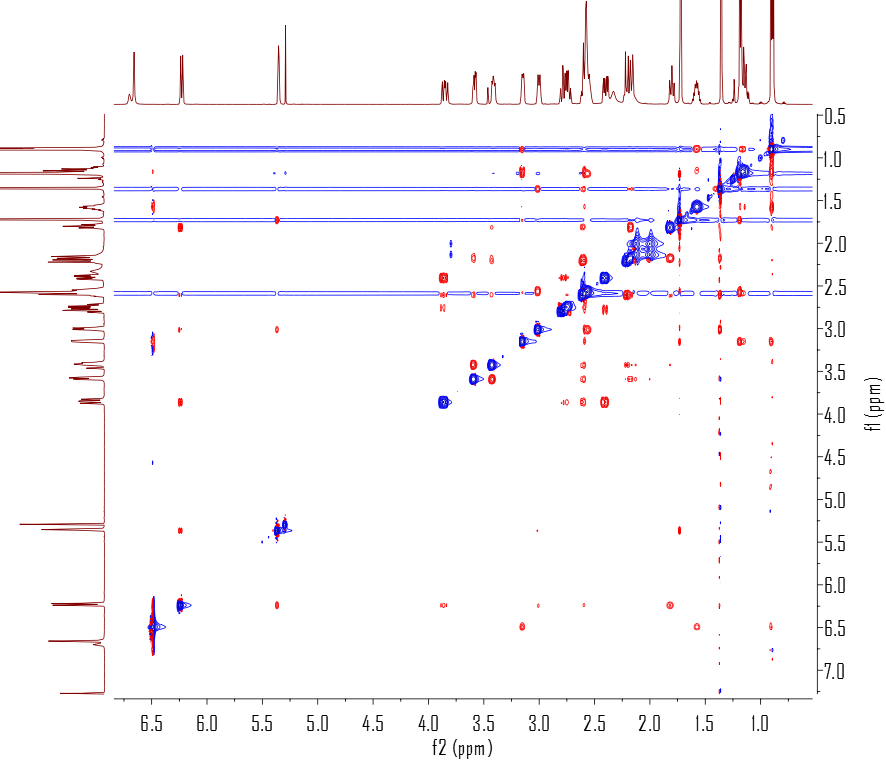


**Figure S4.** HMQC spectrum of **1** (CDCl_3_)


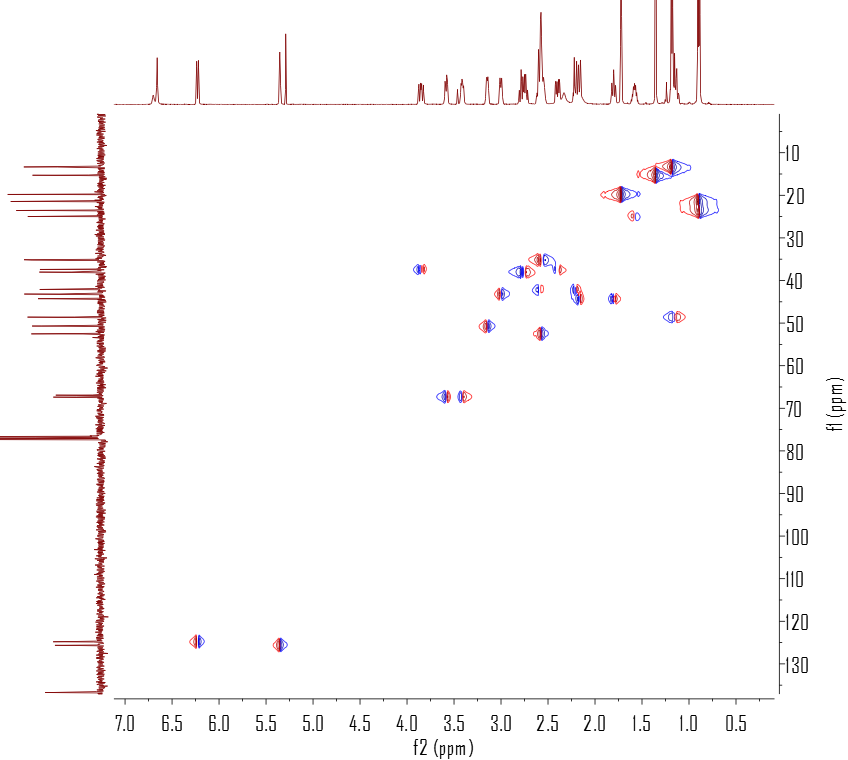


**Figure S5.** HMBC spectrum of **1** (CDCl_3_)


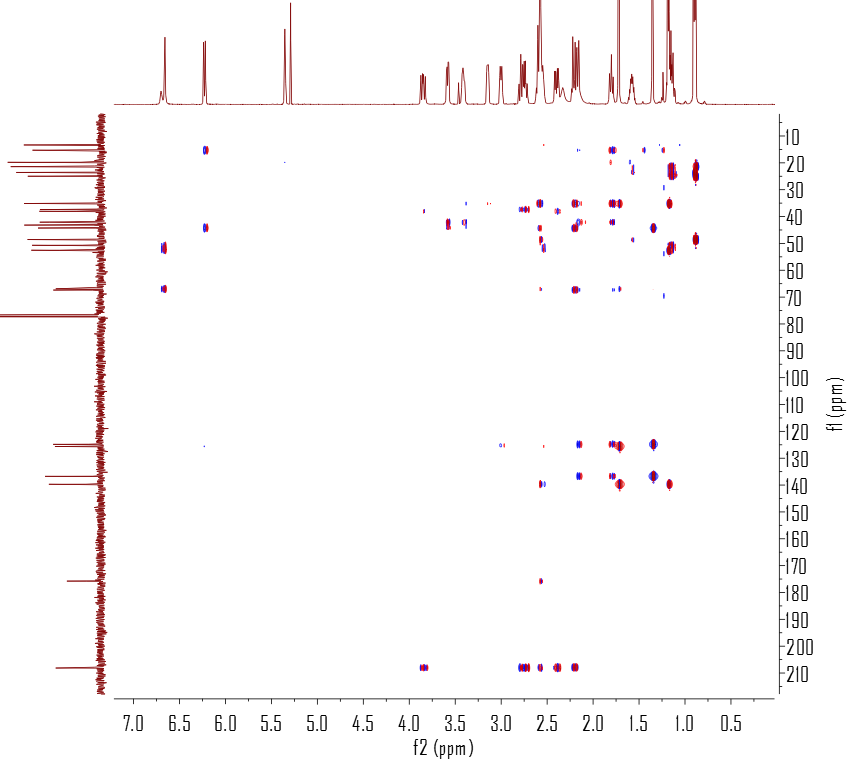


**Figure S6.** NOESY spectrum of **1** (CDCl_3_)


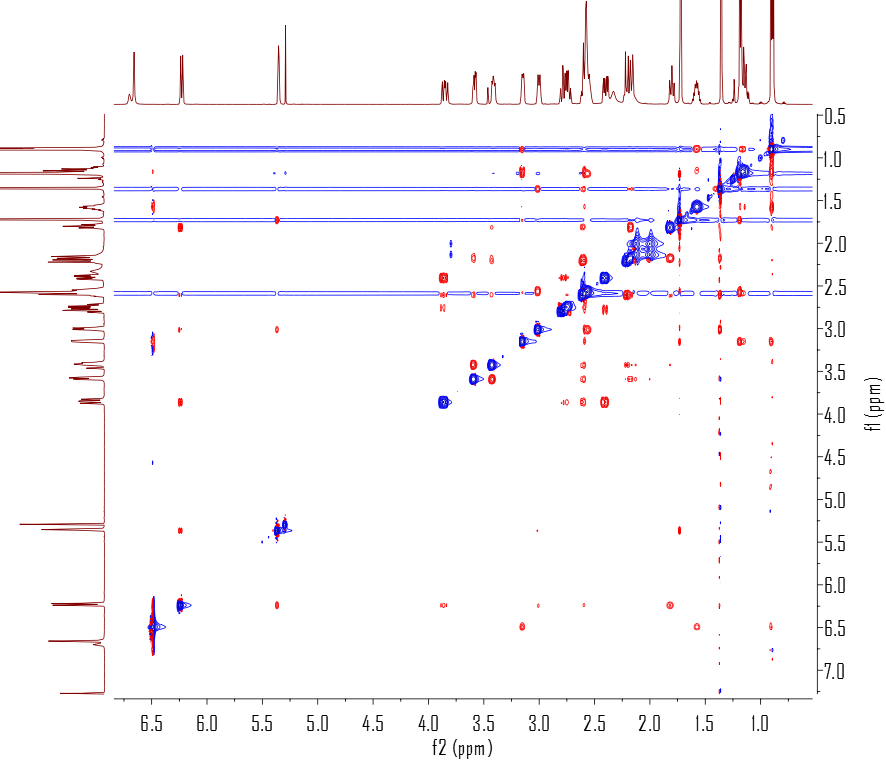


**Figure S7.** HRESIMS spectrum of **1**


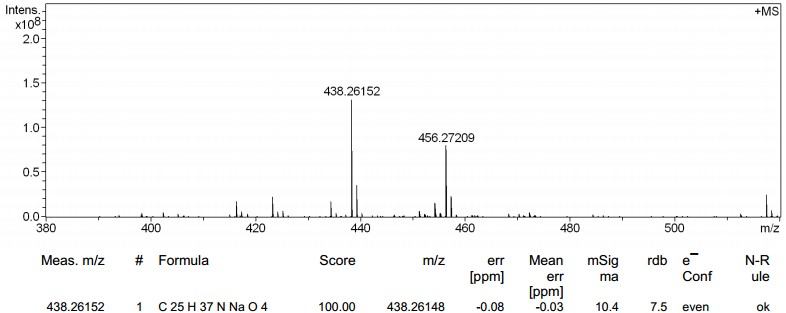


**Figure S8.** IR spectrum of **1**


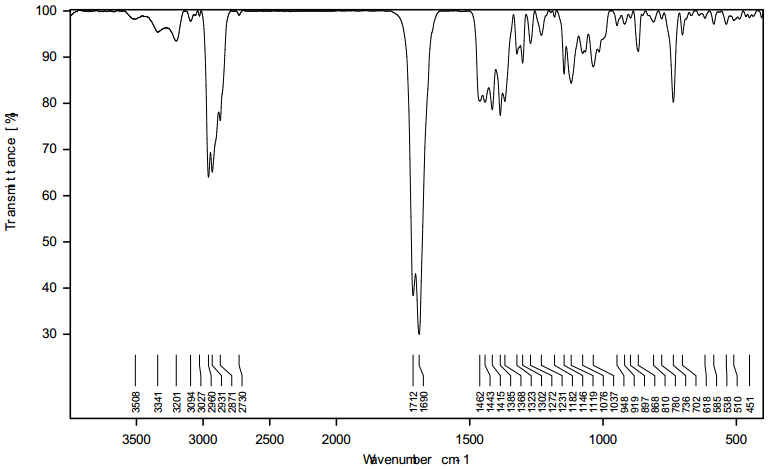


**Figure S9.** UV spectrum of **1** (CH_3_OH)


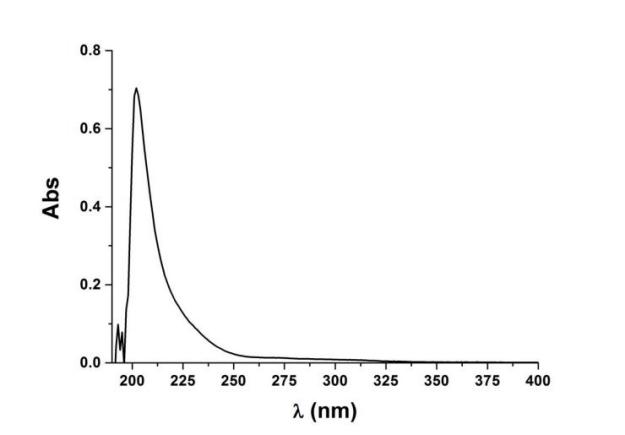


**Figure S10.** ^1^H NMR spectrum of **2** (CDCl_3_, 600 MHz)


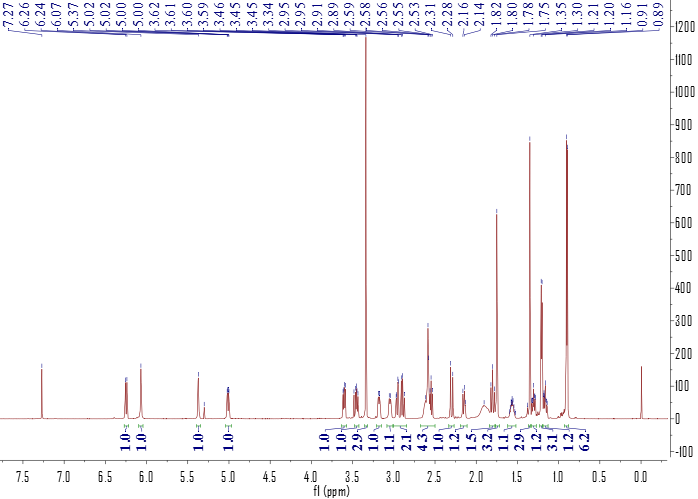


**Figure S11.** ^13^C NMR spectrum of **2** (CDCl_3_, 100 MHz)


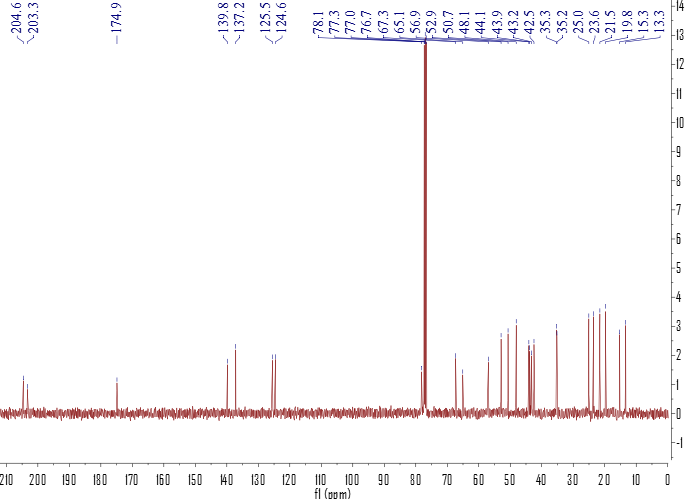


**Figure S12.** COSY spectrum of **2** (CDCl_3_)


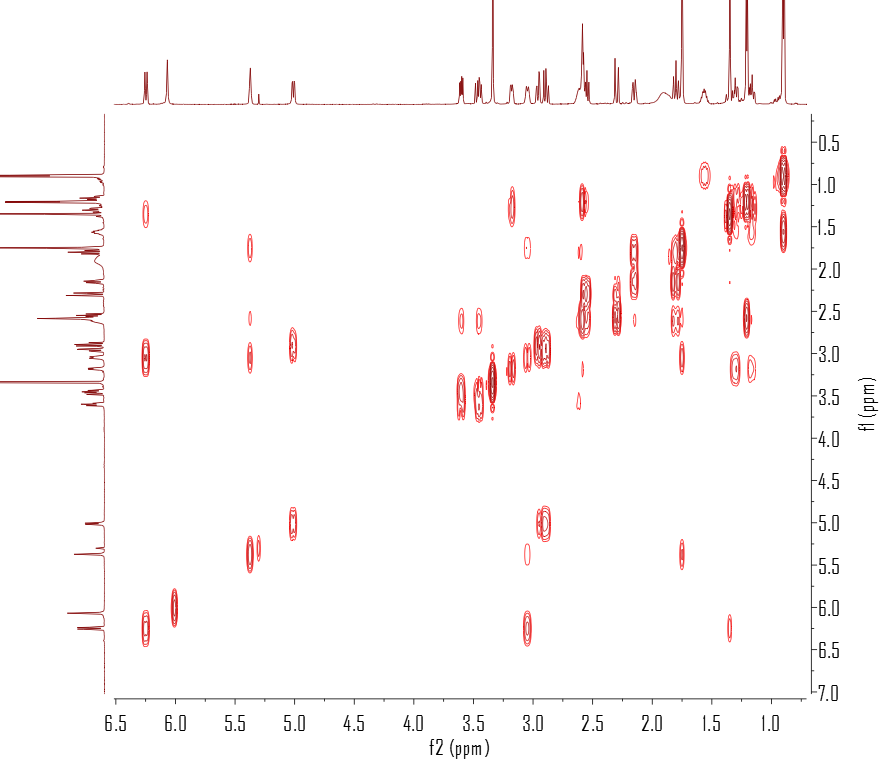


**Figure S13.** HMQC spectrum of **2** (CDCl_3_)


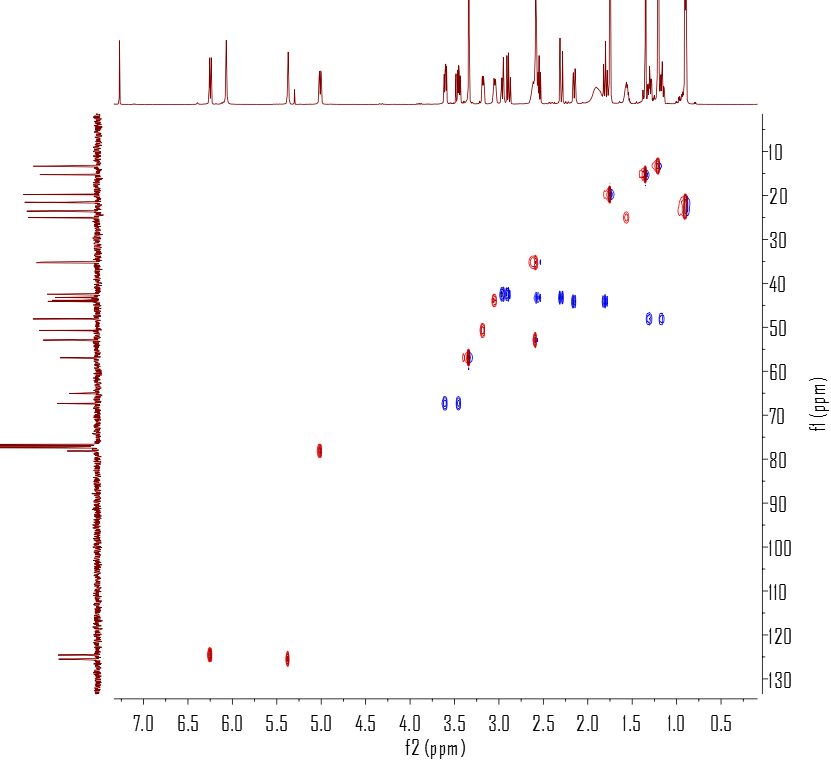


**Figure S14.** HMBC spectrum of **2** (CDCl_3_)


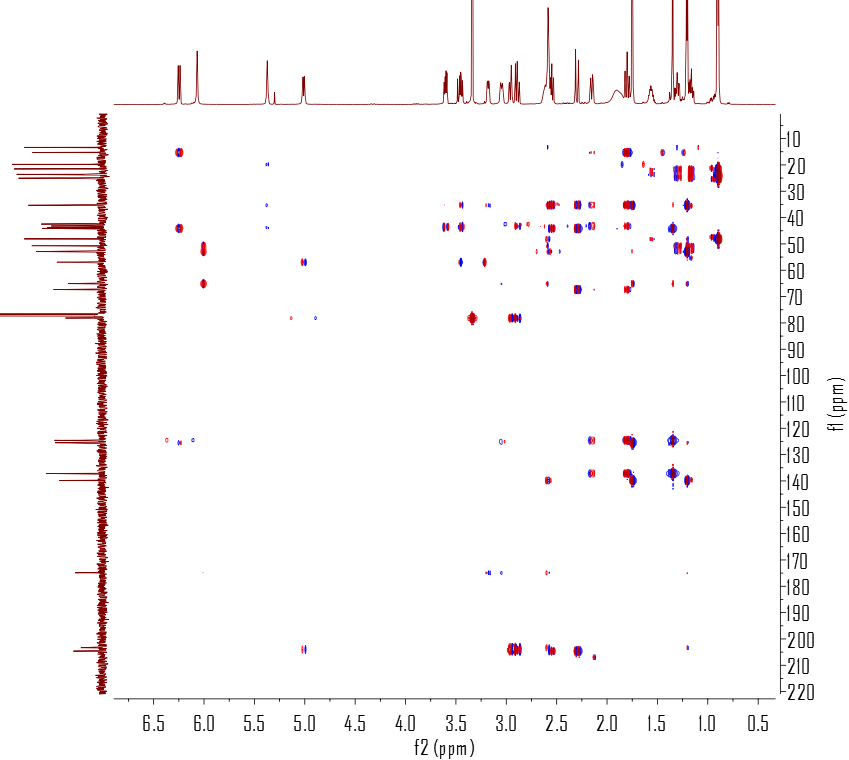


**Figure S15.** NOESY spectrum of **2** (CDCl_3_)


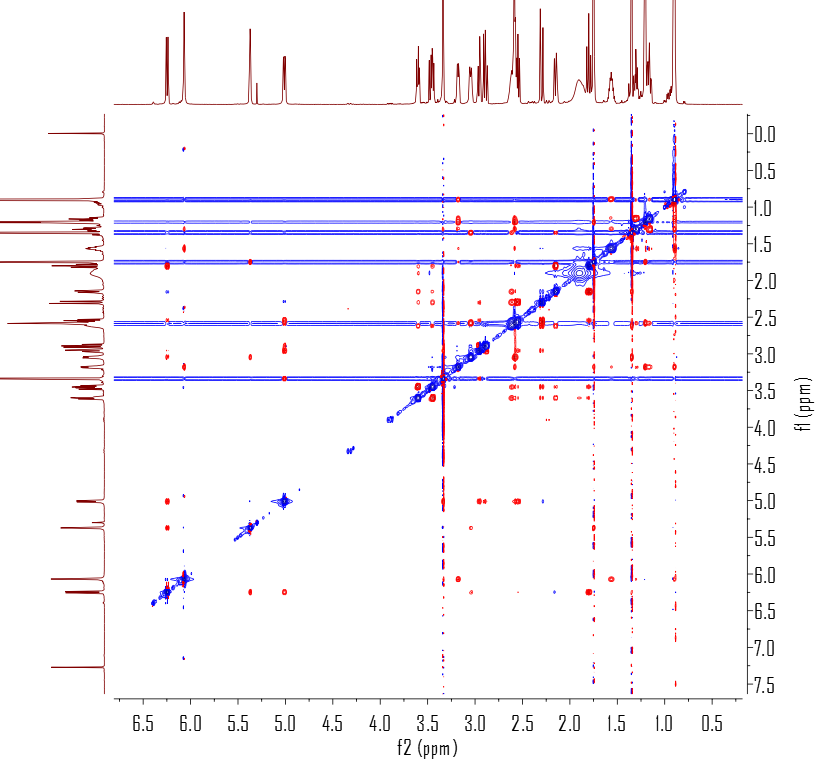


**Figure S16.** HRESIMS spectrum of **2**


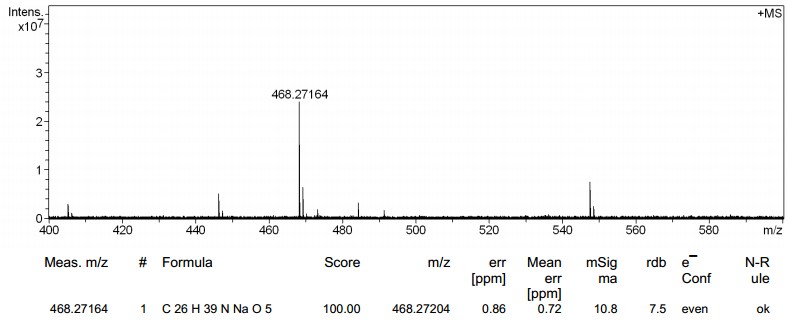


**Figure S17.** IR spectrum of **2**


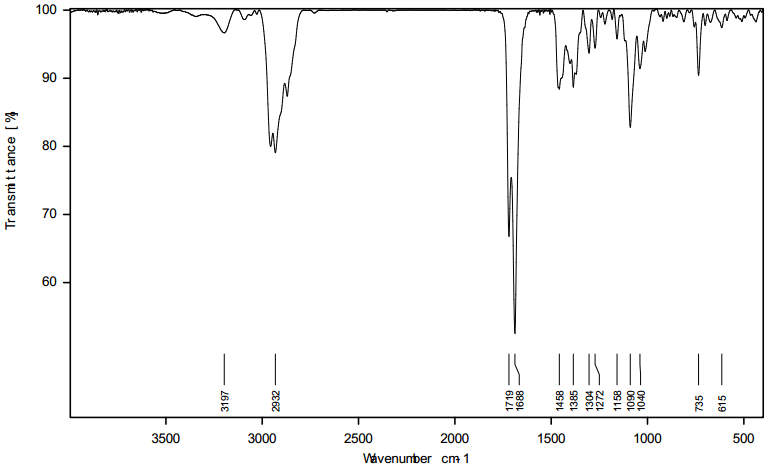


**Figure S18.** UV spectrum of **2** (CH_3_OH)


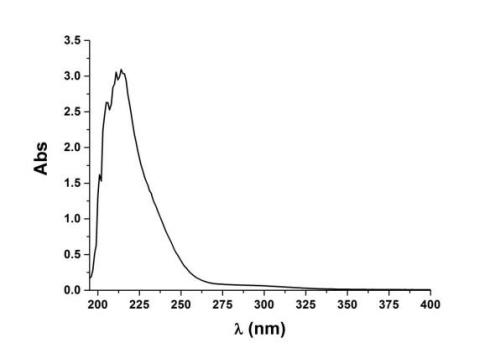


**Figure S19.** ^1^H NMR spectrum of **3** (CDCl_3_, 400 MHz)


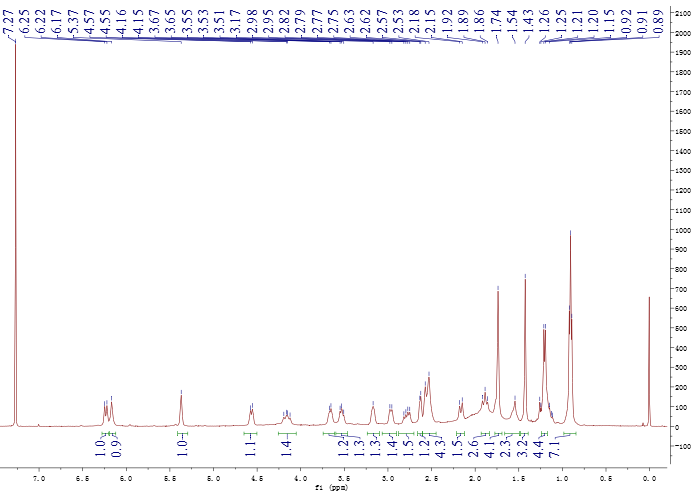


**Figure S20.** ^13^C NMR spectrum of **3** (CDCl_3_, 100 MHz)


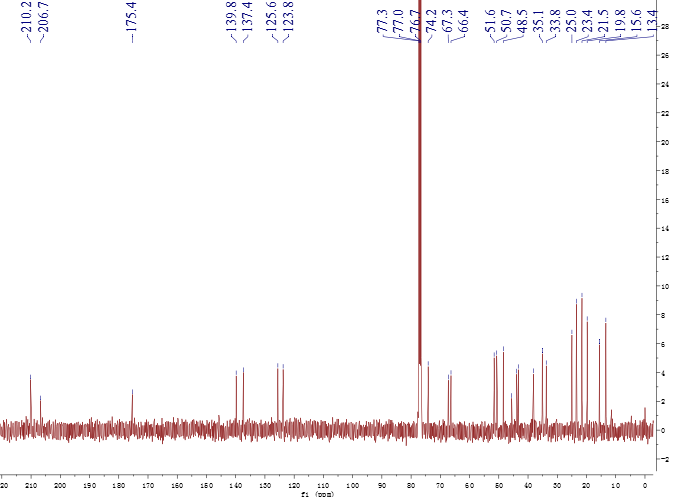


**Figure S21.** COSY spectrum of **3** (CDCl_3_)


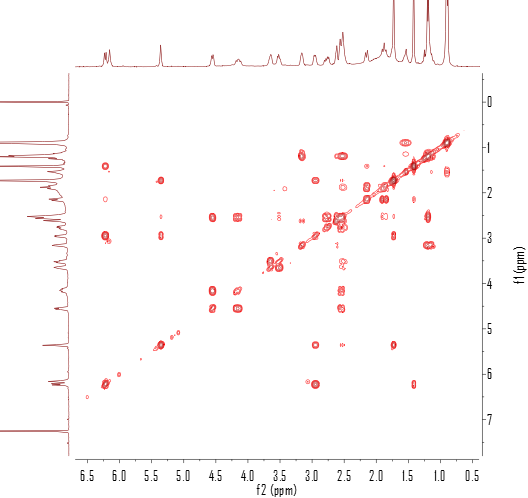


**Figure S22.** HMQC spectrum of **3** (CDCl_3_)


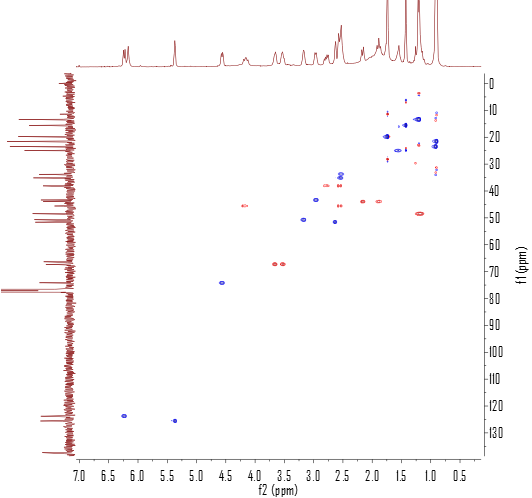


**Figure S23.** HMBC spectrum of **3** (CDCl_3_)


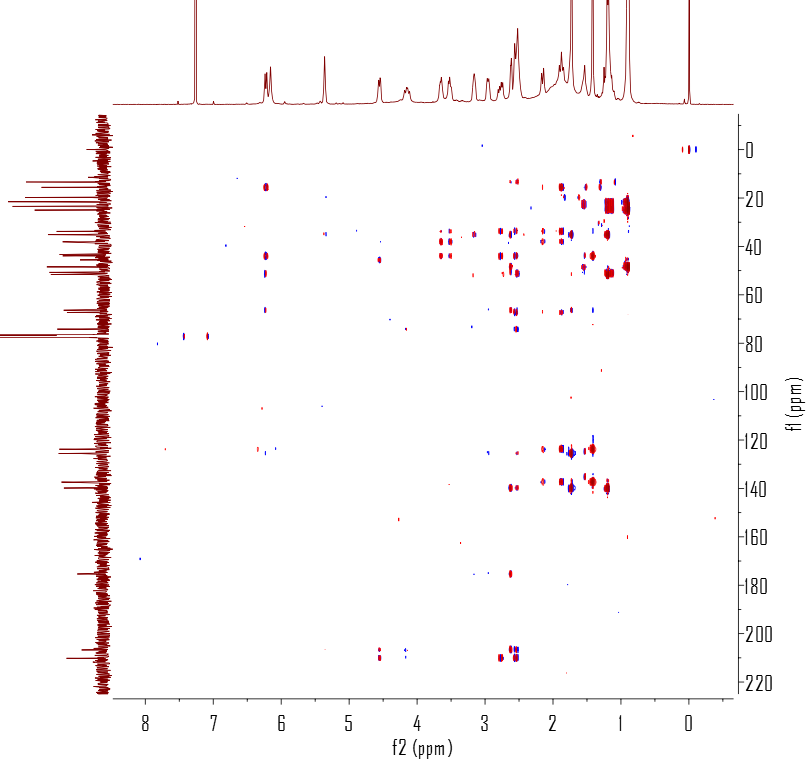


**Figure S24.** NOESY spectrum of **3** (CDCl_3_)


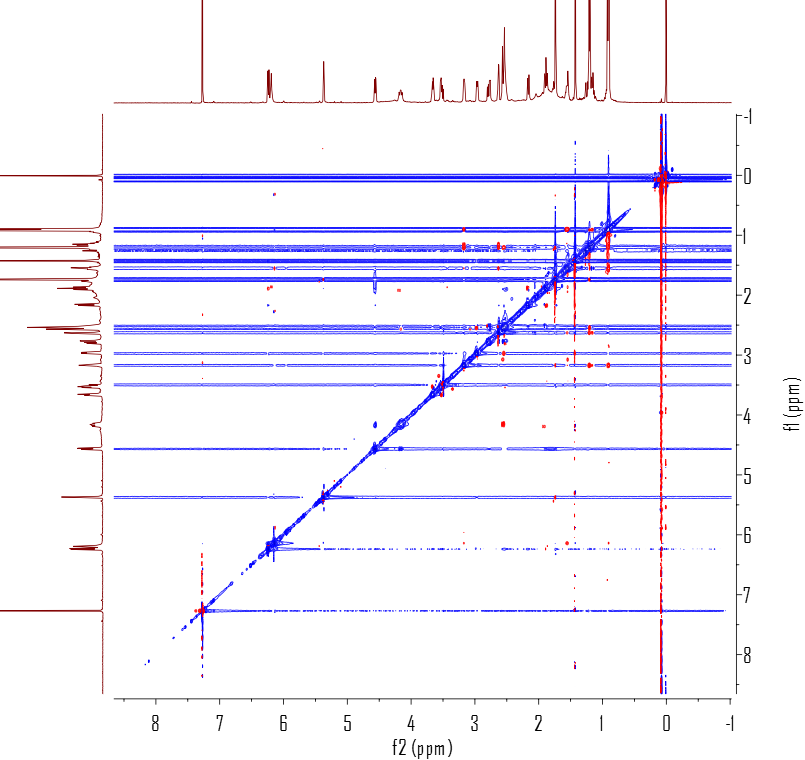


**Figure S25.** HRESIMS spectrum of **3**


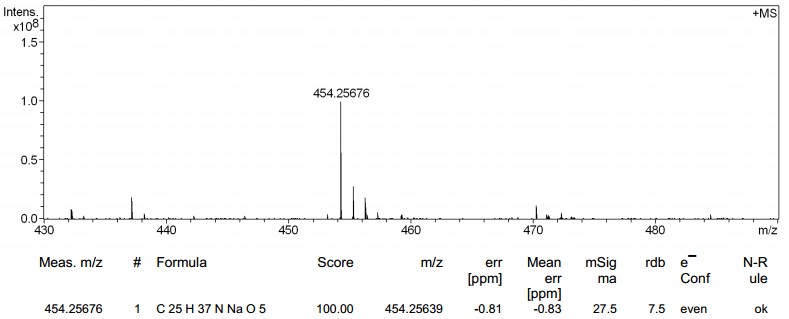


**Figure S26.** IR spectrum of **3**


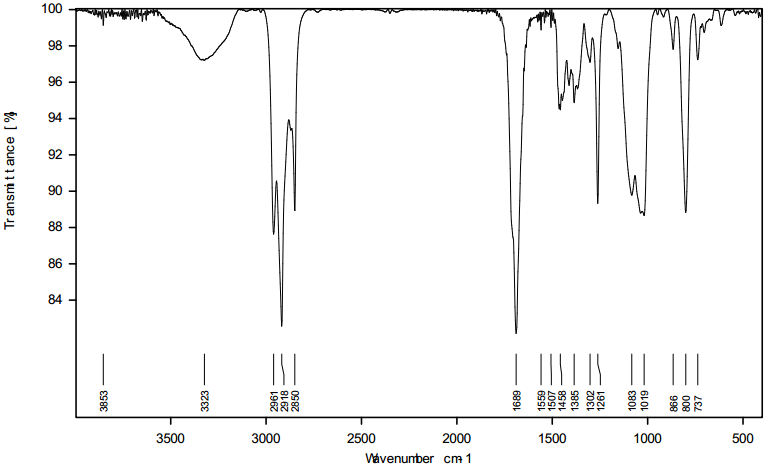


**Figure S27.** UV spectrum of **3** (CH_3_OH)


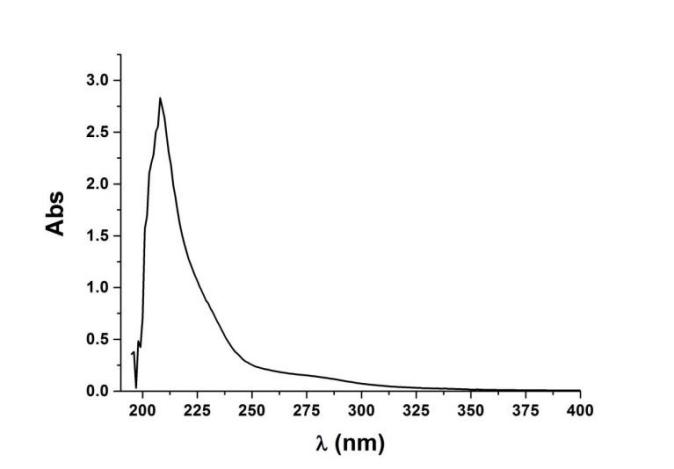


**Figure S28.** ^1^H NMR spectrum of **4** (CDCl_3_, 400 MHz)


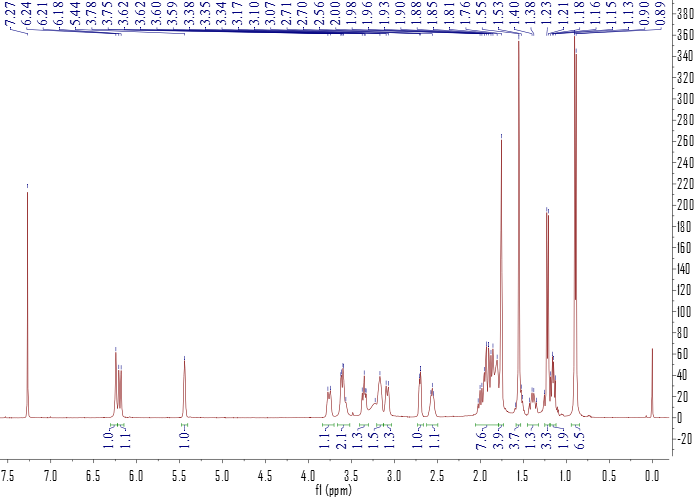


**Figure S29.** ^13^C NMR spectrum of **4** (CDCl_3_, 100 MHz)


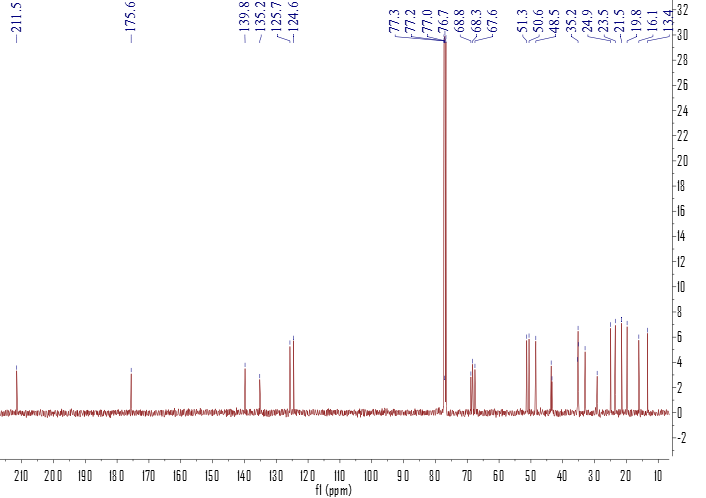


**Figure S30.** COSY spectrum of **4** (CDCl_3_)


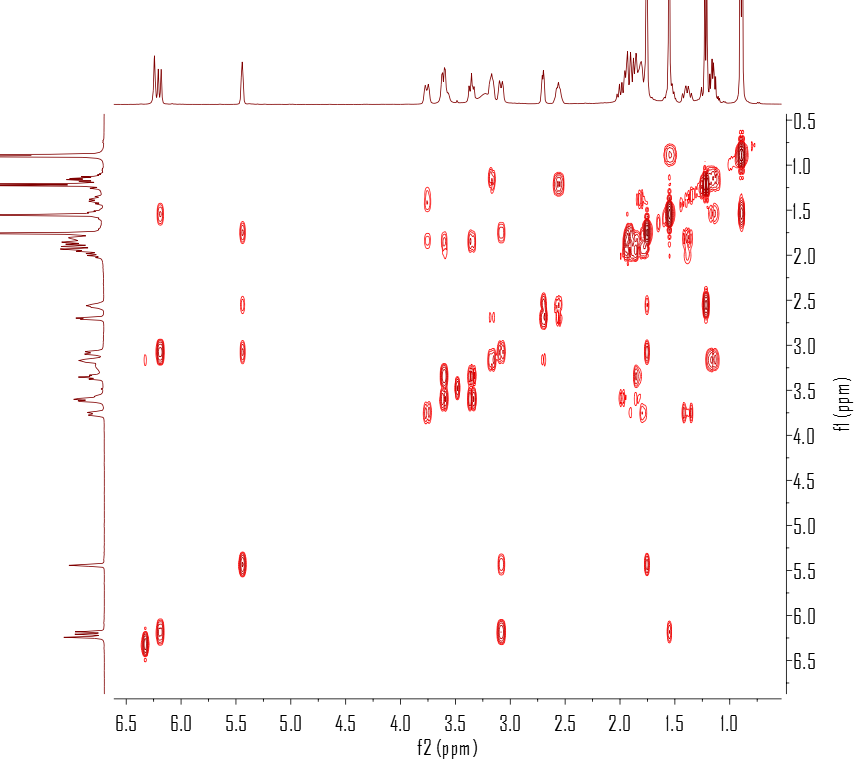


**Figure S31.** HMQC spectrum of **4** (CDCl_3_)


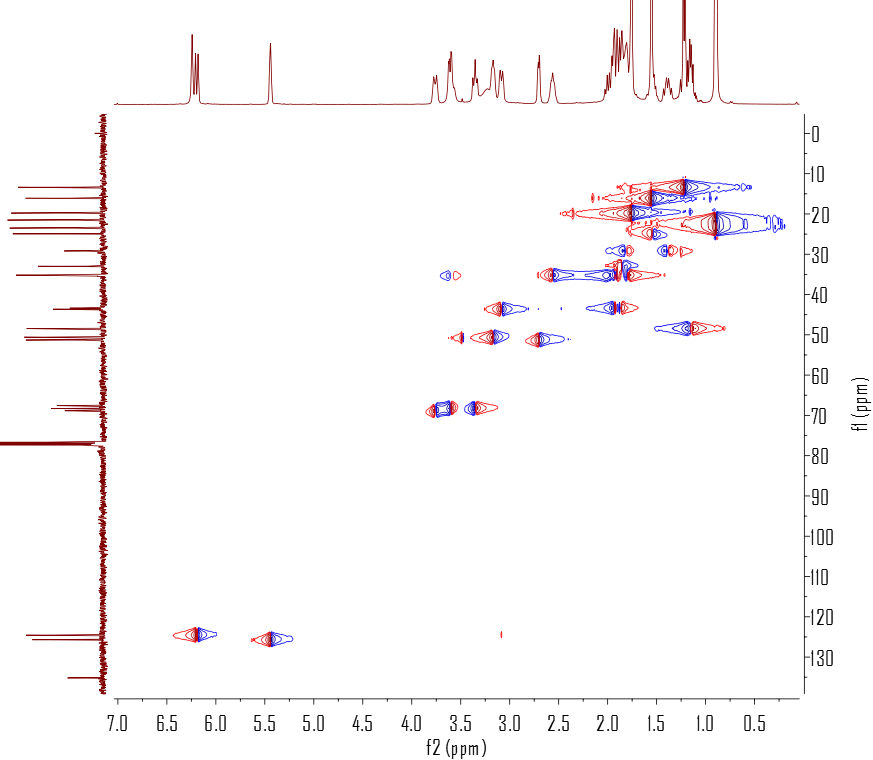


**Figure S32.** HMBC spectrum of **4** (CDCl_3_)


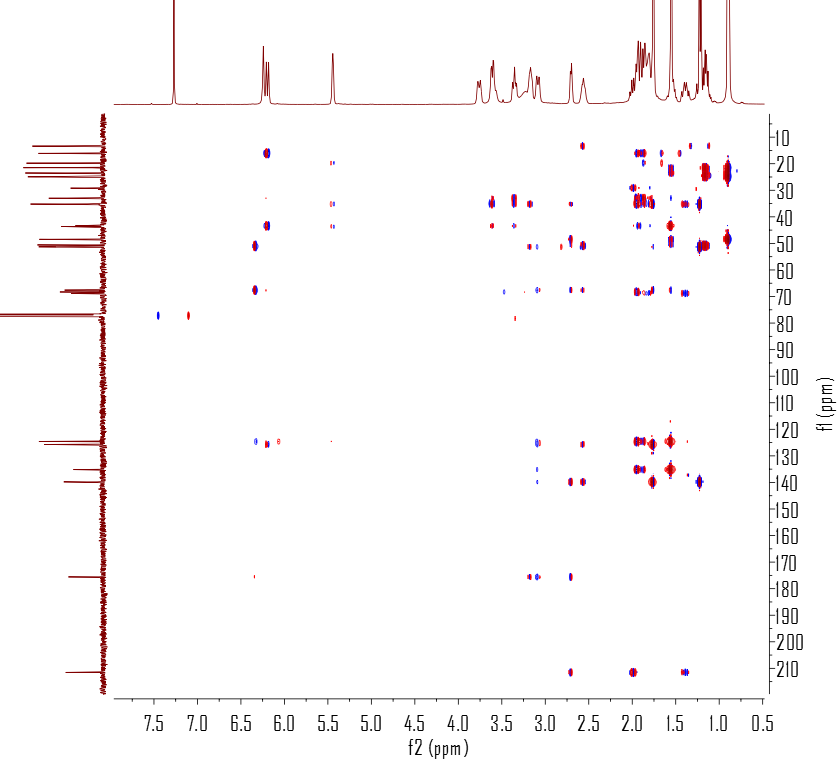


**Figure S33.** NOESY spectrum of **4** (CDCl_3_)


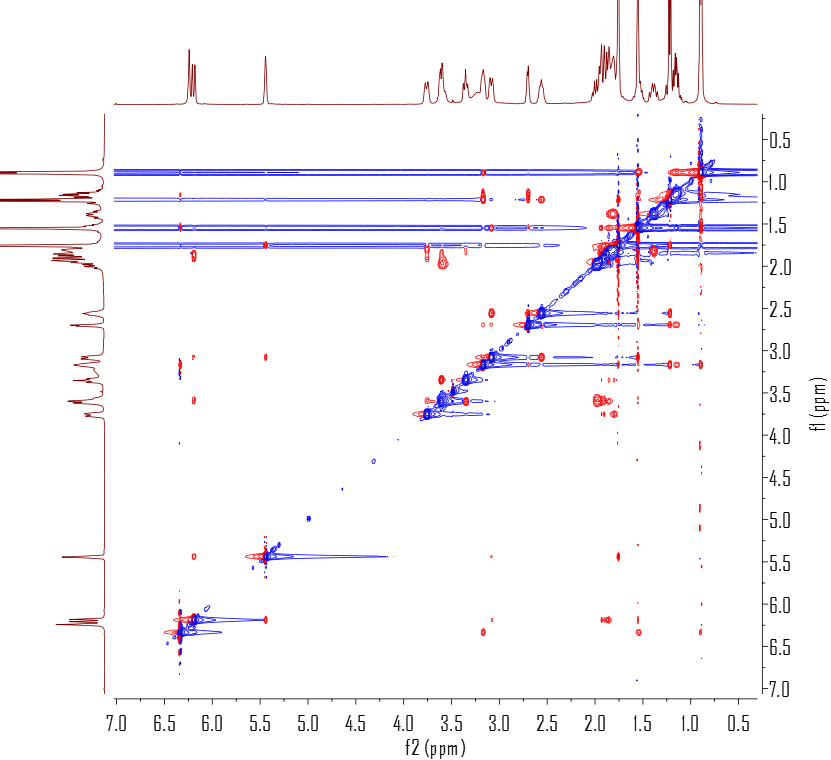


**Figure S34.** HRESIMS spectrum of **4**


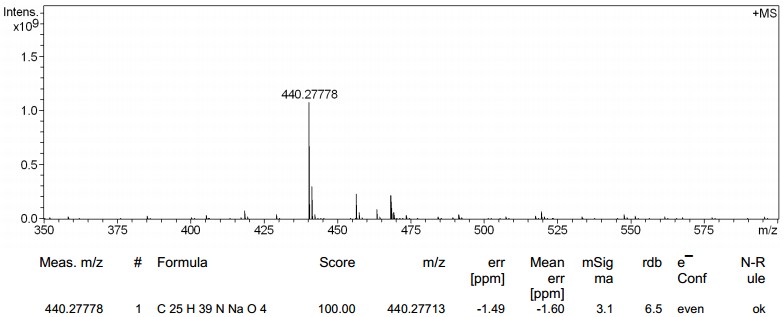


**Figure S35.** IR spectrum of **4**


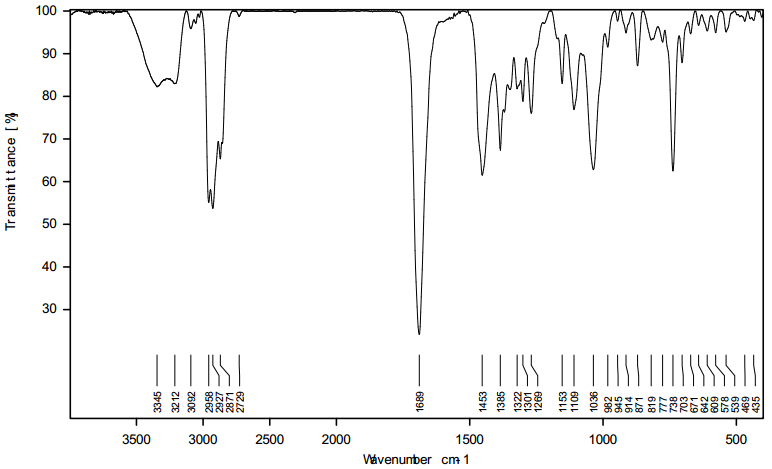


**Figure S36.** UV spectrum of **4** (CH_3_OH)


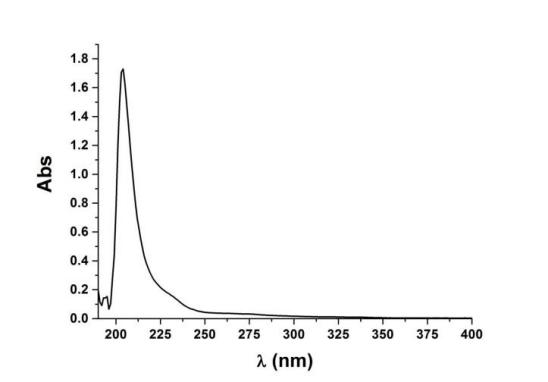


**Figure S37.** ^1^H NMR spectrum of **5** (CDCl_3_, 400MHz)


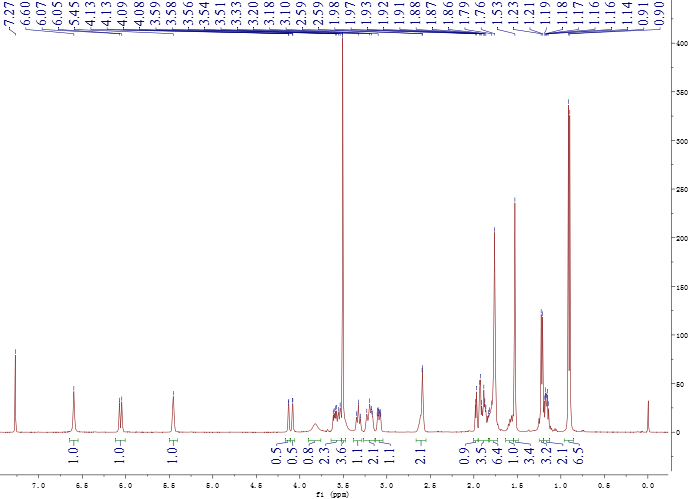


**Figure S38.** ^13^C NMR spectrum of **5** (CDCl_3_, 100 MHz)


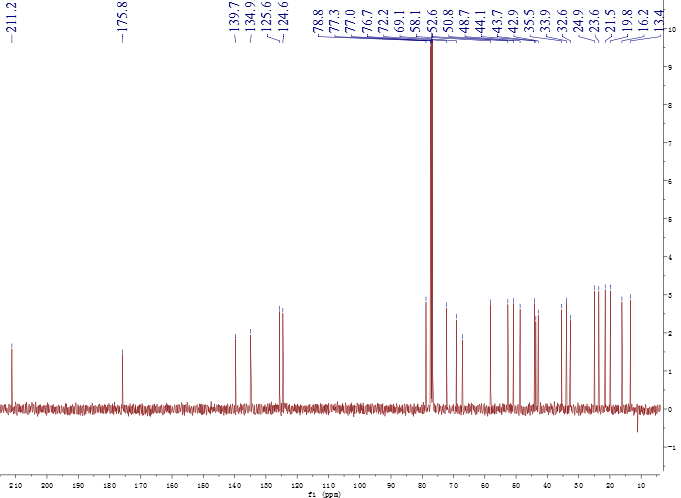


**Figure S39.** COSY spectrum of **5** (CDCl_3_)


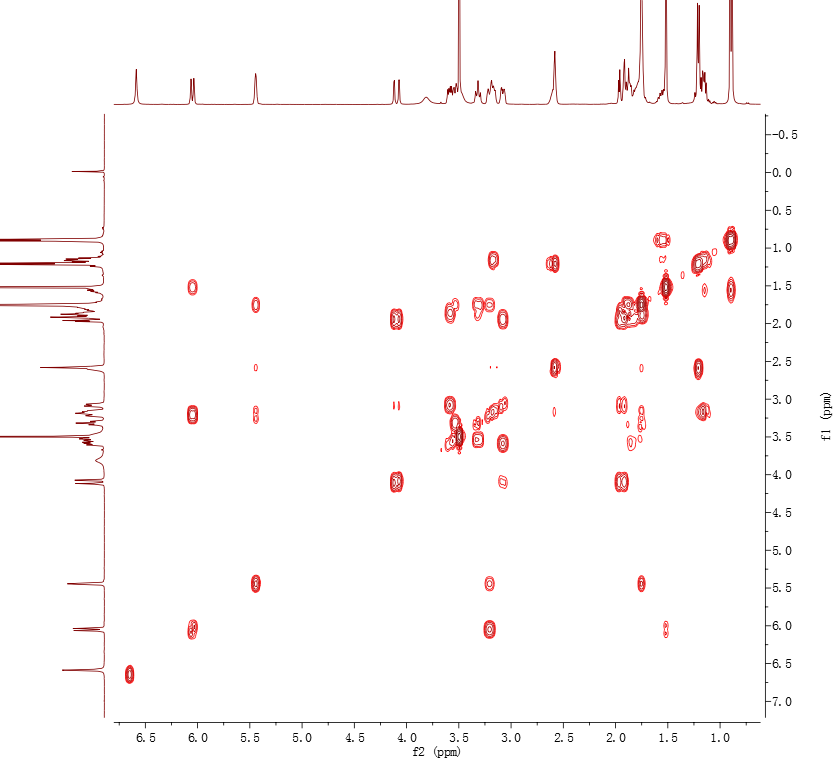


**Figure S40.** HMQC spectrum of **5** (CDCl_3_)


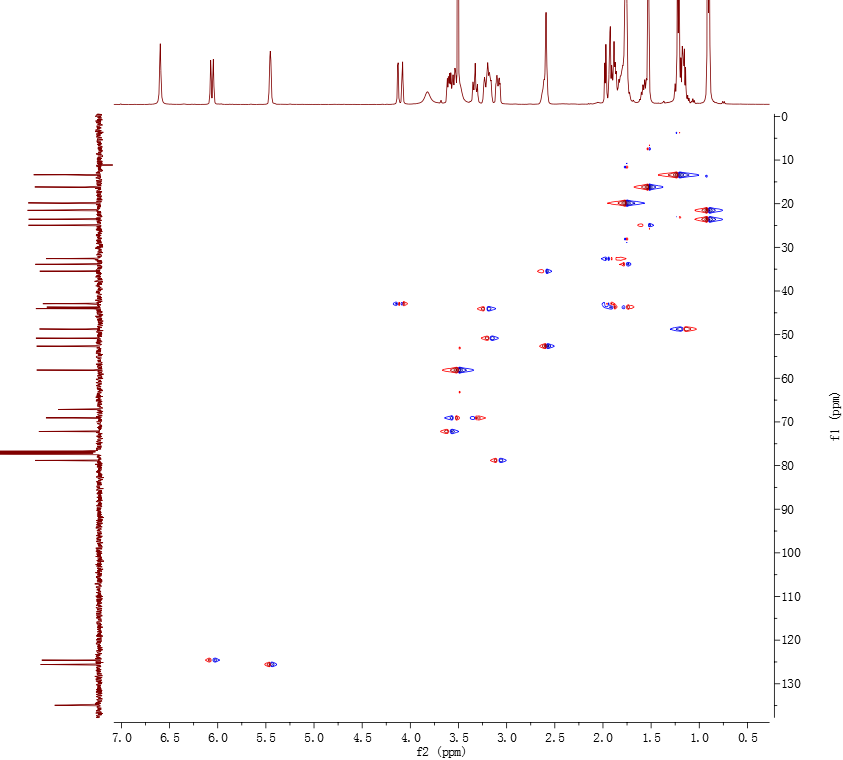


**Figure S41.** HMBC spectrum of **5** (CDCl_3_)


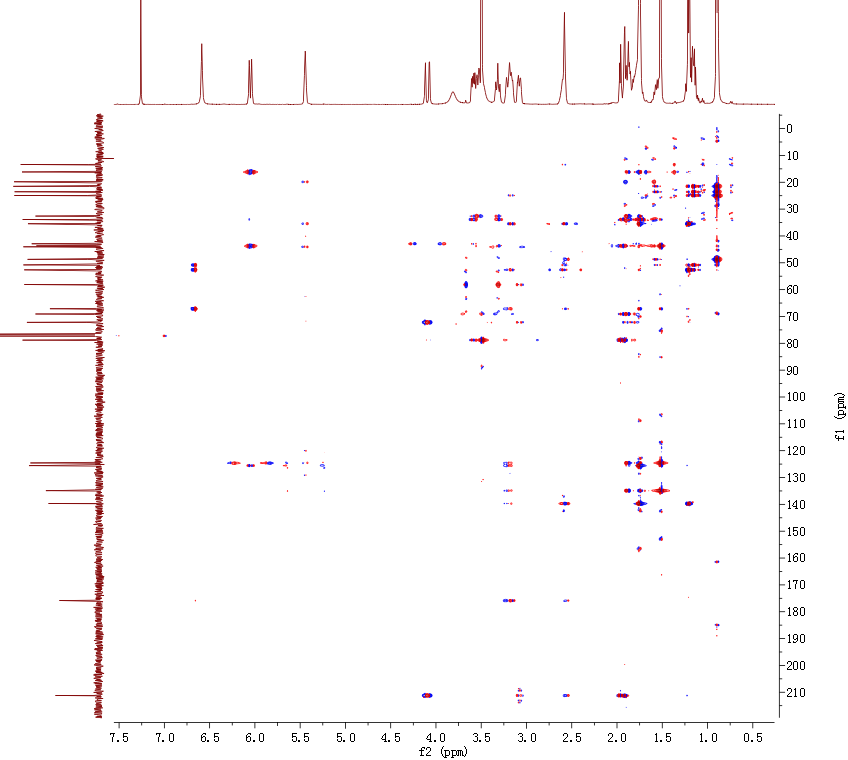


**Figure S42.** NOESY spectrum of **5** (CDCl_3_)


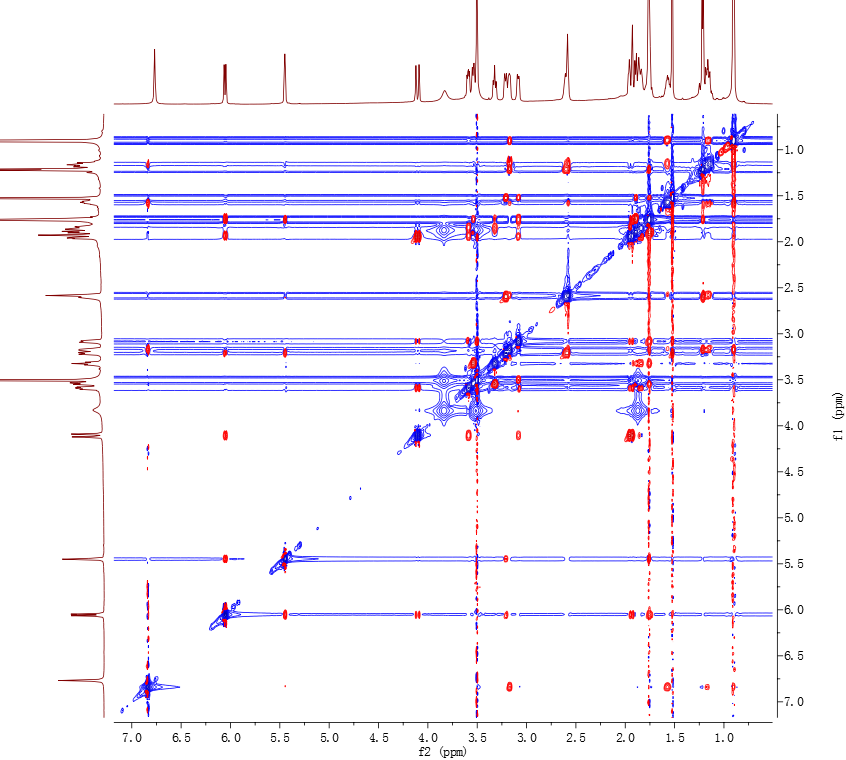


**Figure S43.** HRESIMS spectrum of **5**


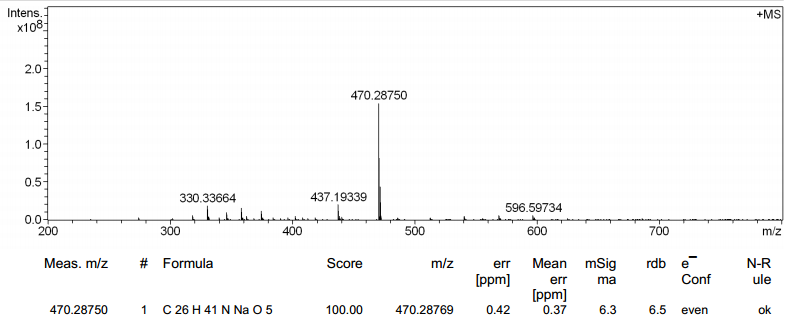


**Figure S44.** IR spectrum of **5**


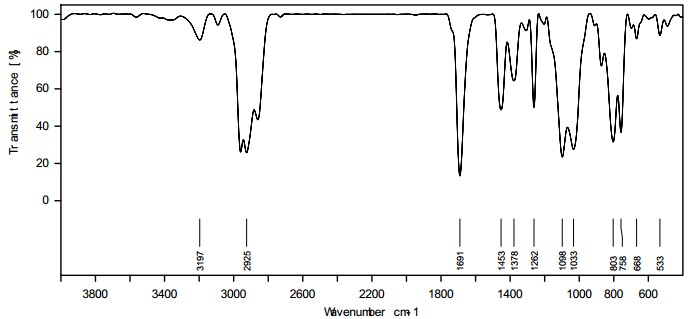


**Figure S45.** UV spectrum of **5**


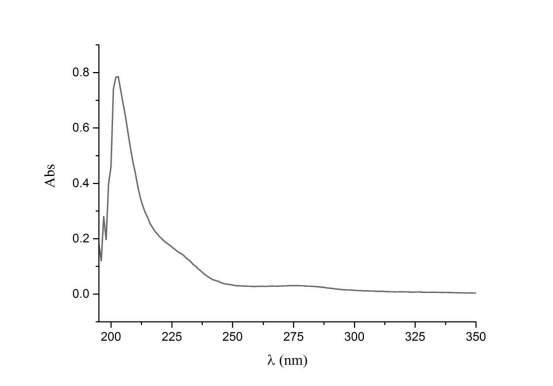


**Figure S46.** ^1^H NMR spectrum of **6** (CDCl_3_, 600 MHz)


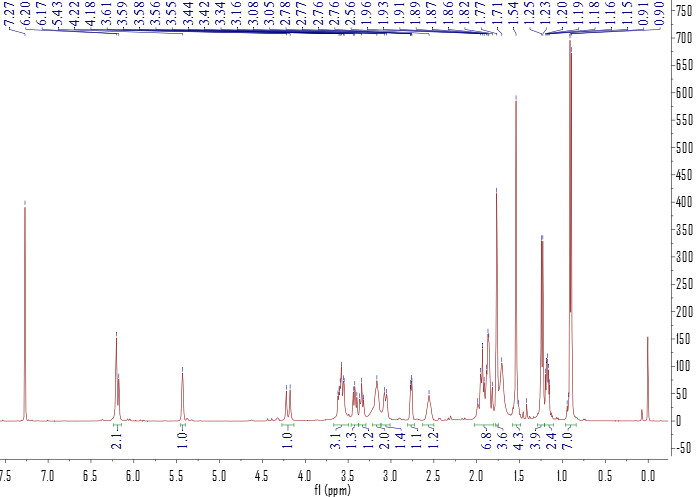


**Figure S47.** ^13^C NMR spectrum of **6** (CDCl_3_, 100 MHz)


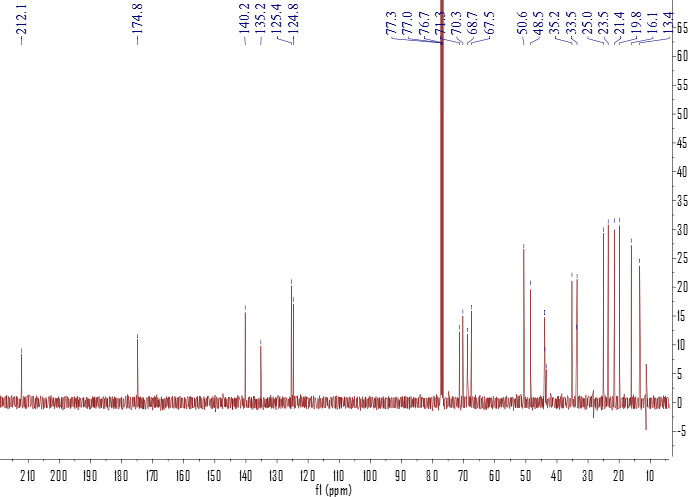


**Figure S48.** COSY spectrum of **6** (CDCl_3_)


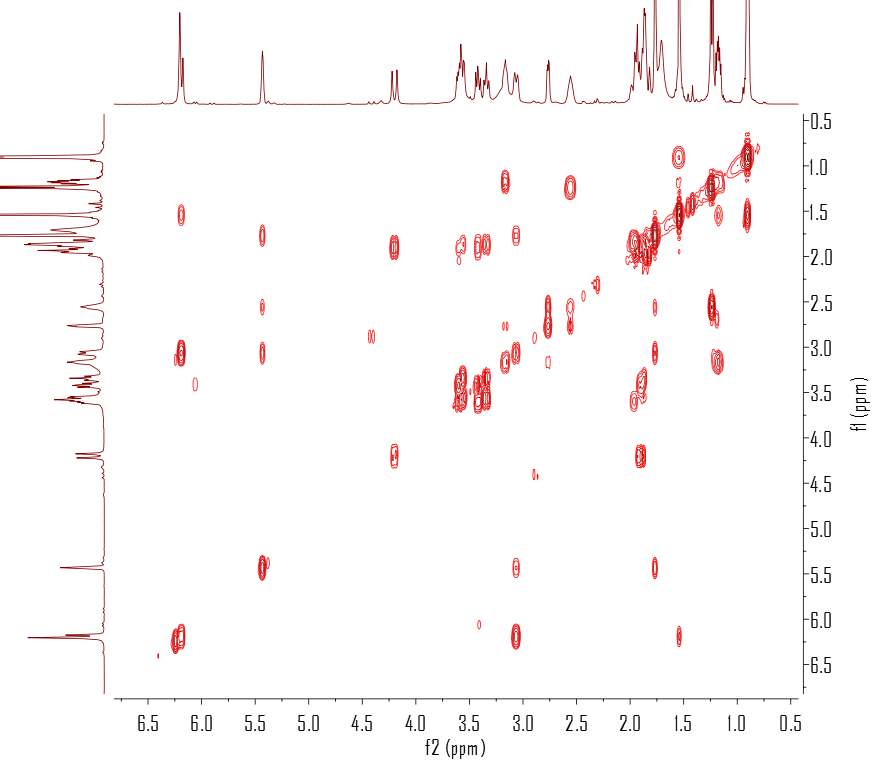


**Figure S49.** HMQC spectrum of **6** (CDCl_3_)


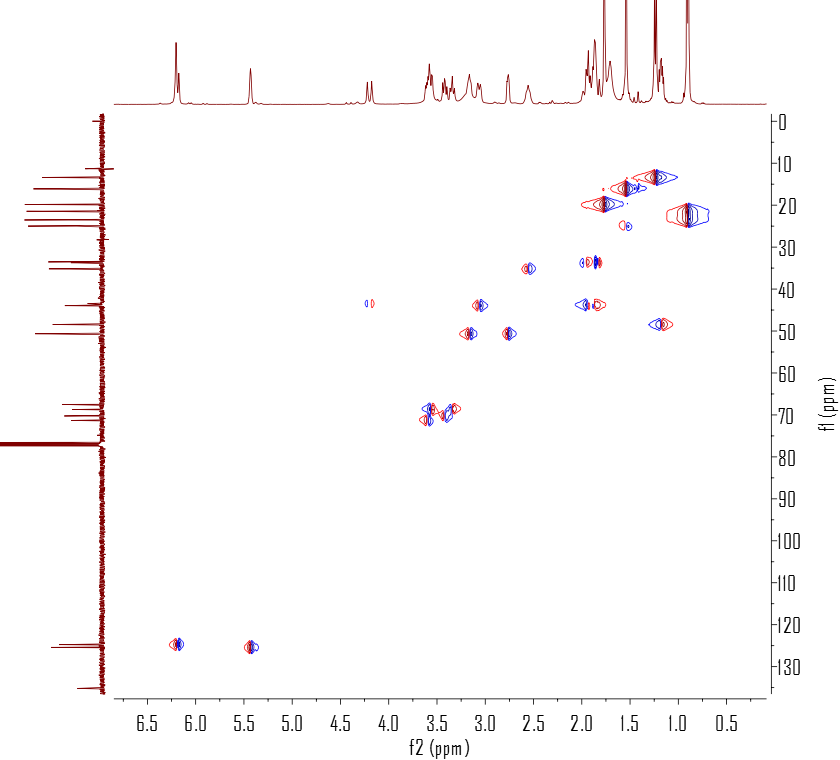


**Figure S50.** HMBC spectrum of **6** (CDCl_3_)


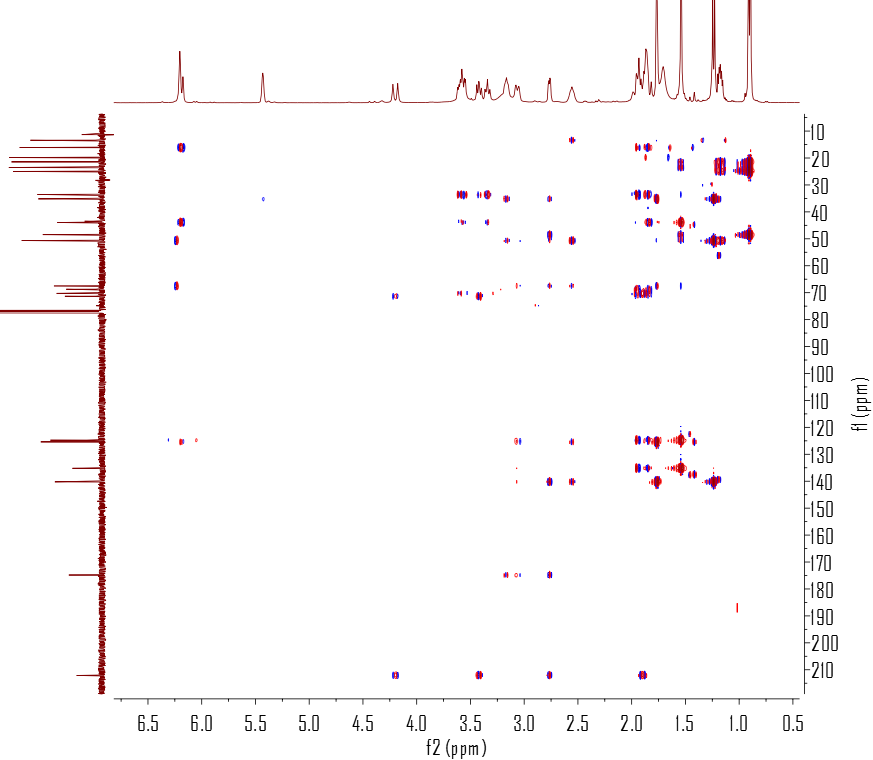


**Figure S51.** NOESY spectrum of **6** (CDCl_3_)


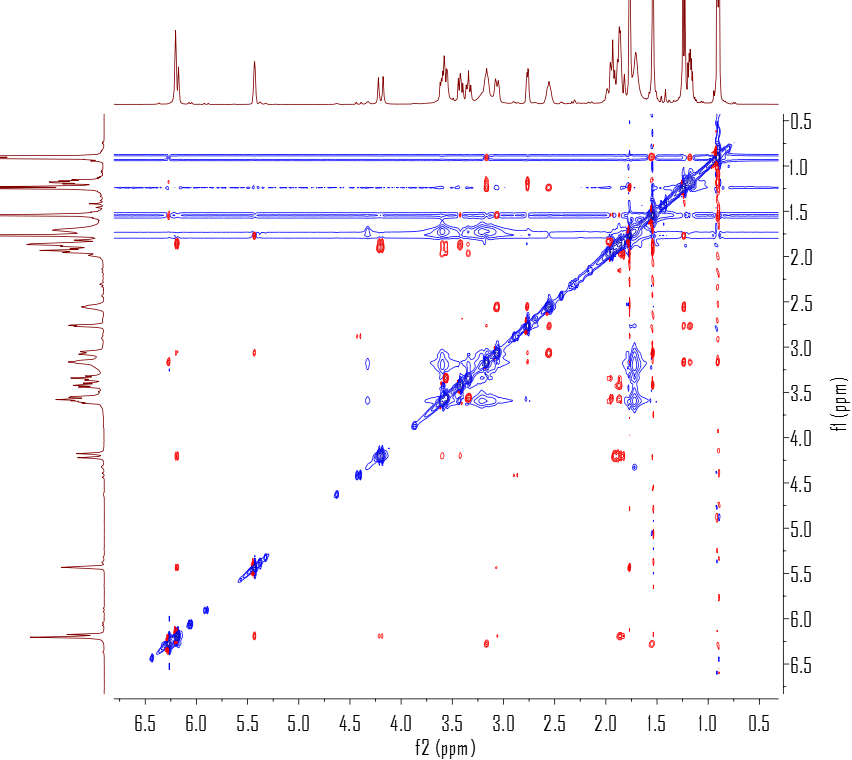


**Figure S52.** HRESIMS spectrum of **6**


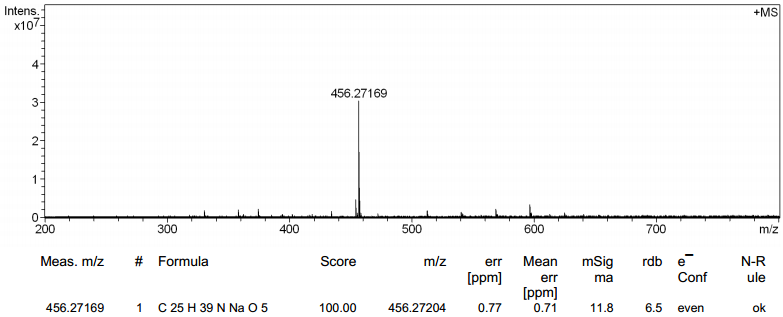


**Figure S53.** IR spectrum of **6**


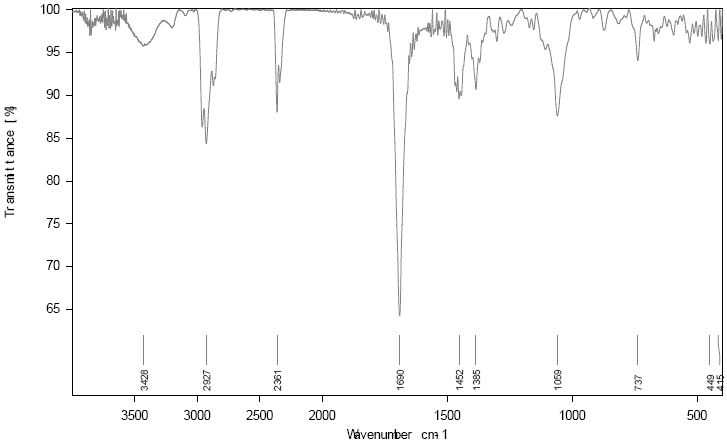


**Figure S54.** UV spectrum of **6** (CH_3_OH)


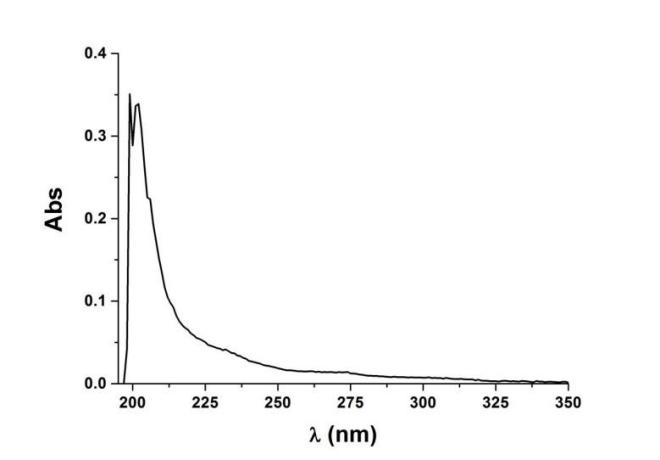


**Figure S55.** ^1^H NMR spectrum of **8** (CDCl_3_, 400 MHz)


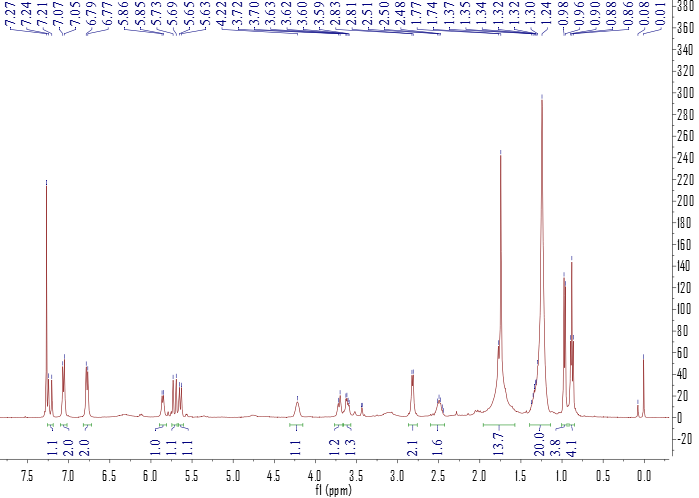


**Figure S56.** ^13^C NMR spectrum of **8** (CDCl_3_, 100 MHz)


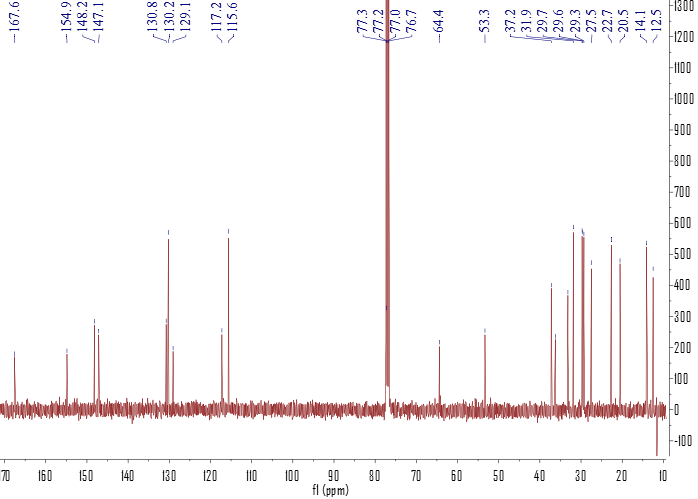


**Figure S57.** COSY spectrum of **8** (CDCl_3_)


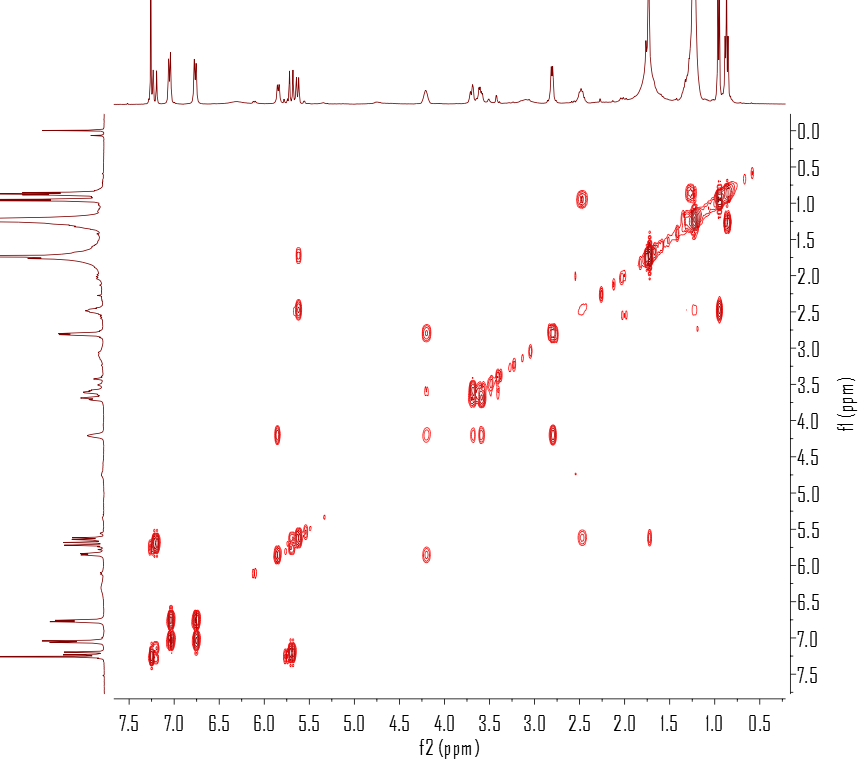


**Figure S58.** HMQC spectrum of **8** (CDCl_3_)


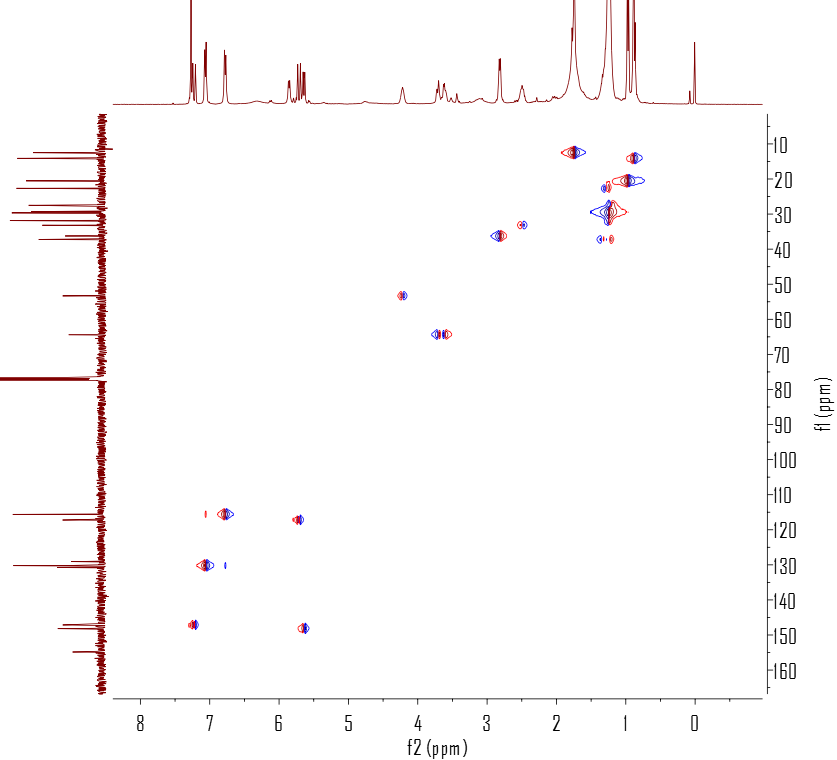


**Figure S59.** HMBC spectrum of **8** (CDCl_3_)


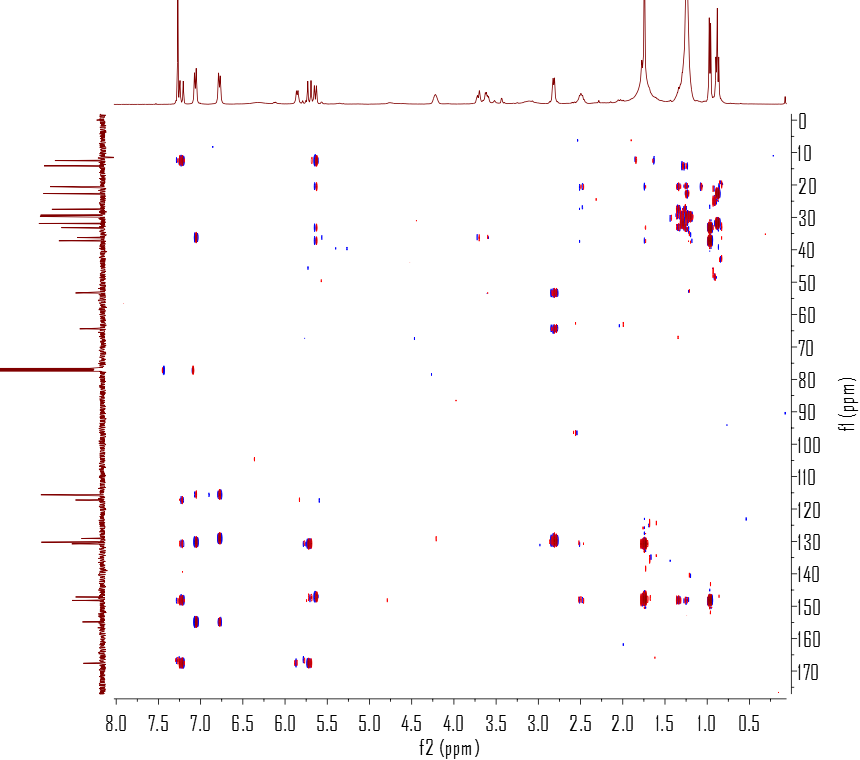


**Figure S60.** HRESIMS spectrum of **8**


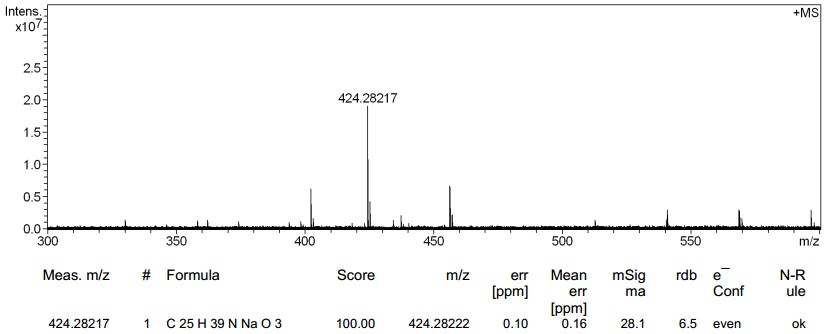


**Figure S61.** IR spectrum of **8**


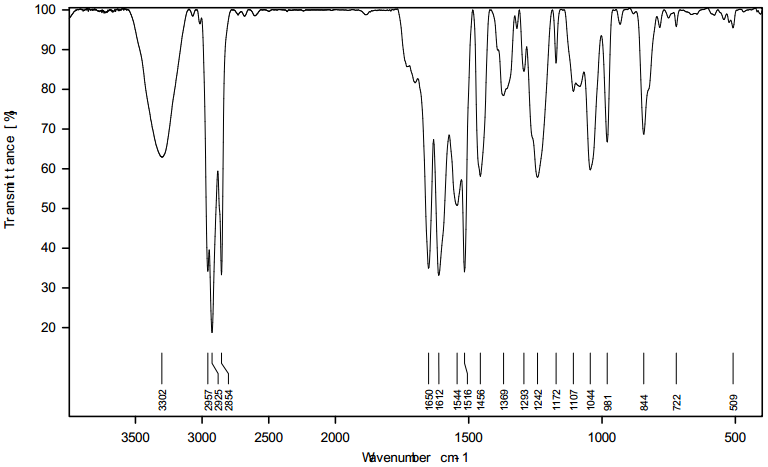


**Figure S62.** UV spectrum of **8** (EtOH)


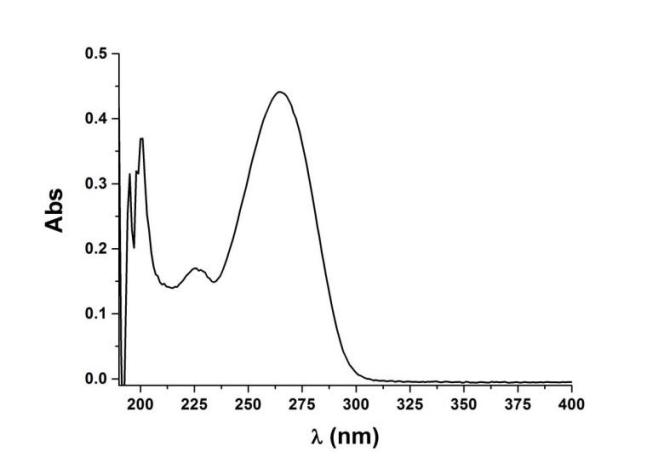


**Table 4.** Antibacterial Activity of Compounds **1**–**9** (MIC, *μ*g/mL)

| Compounds | Gram-positive bacteria | | | | Gram-negative bacteria | | | |
| --- | --- | --- | --- | --- | --- | --- | --- | --- |
|  | *B. subtilis* | *M. luteus* | *B. anthracis* | *S. enterica* | *P. vulgaris* | *S. typhimurium* | *E. coli* | *E. aerogenes* |
| **1** | >100 | >100 | >100 | >100 | 100 | >100 | 100 | 100 |
| **2** | >100 | >100 | 100 | >100 | 100 | 100 | >100 | >100 |
| **3** | >100 | >100 | 100 | >100 | 100 | 100 | 100 | >100 |
| **4** | >100 | >100 | 100 | >100 | 100 | 100 | >100 | >100 |
| **5** | >100 | >100 | 100 | >100 | 100 | 100 | 100 | 100 |
| **6** | >100 | >100 | 100 | >100 | 100 | 100 | >100 | >100 |
| **7** | >100 | >100 | >100 | >100 | 100 | 100 | >100 | >100 |
| **8** | 12.5 | >100 | >100 | >100 | 100 | 100 | 100 | >100 |
| **9** | >100 | >100 | 100 | >100 | 100 | 100 | 100 | 100 |
| Ciprofloxacin | 0.78125 | 0.78125 | 0.78125 | 0.78125 | 0.78125 | 0.78125 | 0.78125 | 0.78125 |

**Table 5.** Cytotoxic Activity of Compounds **1**–**9** (IC_50_, *μ*M)

| Compounds | MCF-7 | A549 | HepG2 | HT-29 | SGC-7901 |
| --- | --- | --- | --- | --- | --- |
| **1** | >100 | >100 | >100 | >100 | >100 |
| **2** | >100 | >100 | >100 | >100 | >100 |
| **3** | >100 | >100 | >100 | >100 | >100 |
| **4** | >100 | >100 | >100 | 49.09 | >100 |
| **5** | 83.71 | 61.28 | 62.43 | 55.31 | 56.83 |
| **6** | >100 | >100 | >100 | 55.48 | >100 |
| **7** | 25.63 | 58.70 | 57.01 | 29.92 | 65.49 |
| **8** | 48.13 | 62.91 | 42.26 | 49.31 | 34.68 |
| **9** | >100 | >100 | >100 | >100 | >100 |
| 5-Fu | 63.98 | 66.82 | 58.10 | 67.13 | >100 |

**Table 6.** X-ray crystallographic data of compound **1**

| Empirical formula | C_25_H_37_NO_4_ |
| --- | --- |
| Formula weight | 415.55 |
| Temperature/K | 293 |
| Crystal system | monoclinic |
| Space group | P21 |
| a/Å | 9.3417(2) |
| b/Å | 10.9357(3) |
| c/Å | 24.5671(6) |
| α/° | 90 |
| β/° | 91.595(2) |
| γ/° | 90 |
| Volume/Å^3^ | 2508.75(11) |
| Z | 4 |
| ρ_calc_g/cm^3^ | 1.100 |
| μ/mm^‑1^ | 0.073 |
| F(000) | 904.0 |
| Radiation | Mo Kα (λ = 0.71073) |
| 2Θ range for data collection/° | 6.566 to 52.744 |
| Index ranges | -11 ≤ h ≤ 11, -13 ≤ k ≤ 13, -30 ≤ l ≤ 30 |
| Reflections collected | 34389 |
| Independent reflections | 10235 [R_int_ =0.0375, R_sigma_ = 0.0473] |
| Data/restraints/parameters | 1235/1/553 |
| Goodness-of-fit on F^2^ | 1.031 |
| Final R indexes [I>=2σ (I)] | R_1_ = 0.0562, wR_2_ = 0.1332 |
| Final R indexes [all data] | R_1_ = 0.0834, wR_2_ = 0.1500 |
| Largest diff. peak/hole / e Å^-3^ | 0.3/-0.2 |
| Flack parameter | 0.1(4) |

**S1. Computation Section**

**1.** **The absolute configuration of C-19 in 2 was determined by calculation of the electronic circular dichroism (ECD).**


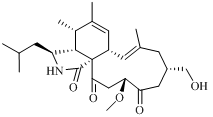

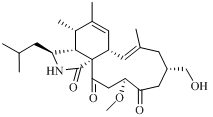


(3*S**,4*R**,5*S**,8*S**,9*S**,16*S**,19*R*)-**2** (**2a**) (3*S**,4*R**,5*S**,8*S**,9*S**,16*S**,19*S*)-**2** (**2b**)

Compound **2** had two possible structures: (3*S**,4*R**,5*S**,8*S**,9*S**,16*S**,19*R*)-**2** (**2a**) and (3*S**,4*R**,5*S**,8*S**,9*S**,16*S**,19*S*)-**2** (**2b**). Overall, conformation searching on **2a** and **2b** by the MMFF94s force field gave 12 and 10 conformers with population higher than 1%, respectively (Figures S63 and S64). All of these conformers were optimized at the B3LYP/6-31G(d,p) of theory to give ten and nine conformers within a 3 kcal/mol energy threshold from global minimum, respectively. These predominant conformers were subjected to theoretical calculation of ECD using time-dependent density functional theory (TDDFT) at B3LYP/6-31G(d,p) level in gas phase. The calculated ECD curves for **2a** and **2b** and weighted ECD were all generated using SpecDis 1.60 with σ = 0.3 eV, and UV shift 7 nm, respectively. As shown in Figure S65, the calculated ECD spectrum of **2b** matched well with the experimental one, suggesting the absolute configuration of **C-19** to be *S*.


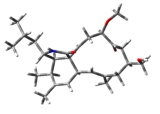

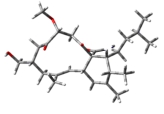

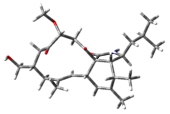


**2a-1 2a-2 2a-3**


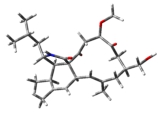

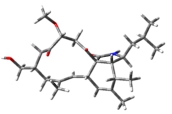

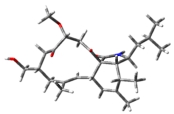


**2a-4 2a-5 2a-6**


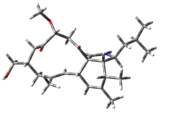

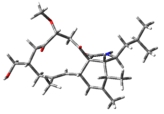

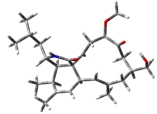


**2a-7 2a-8 2a-9**


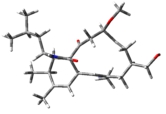

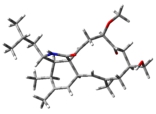


**2a-10 2a-11**

**Figure S63**. Optimized conformers of **2a** (**2a-1**-**2a-11**).


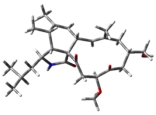

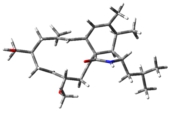

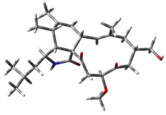


**2b-1 2b-2 2b-3**


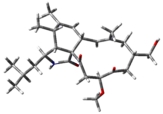

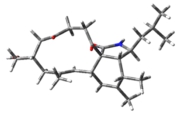

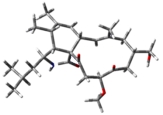


**2b-4 2b-5 2b-6**


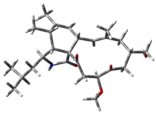

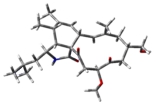

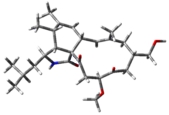


**2b-7 2b-8 2b-9**


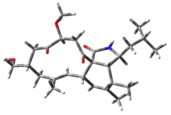


**2b-10**

**Figure S64**. Optimized conformers of **2b** (**2b-1-2b-10**).


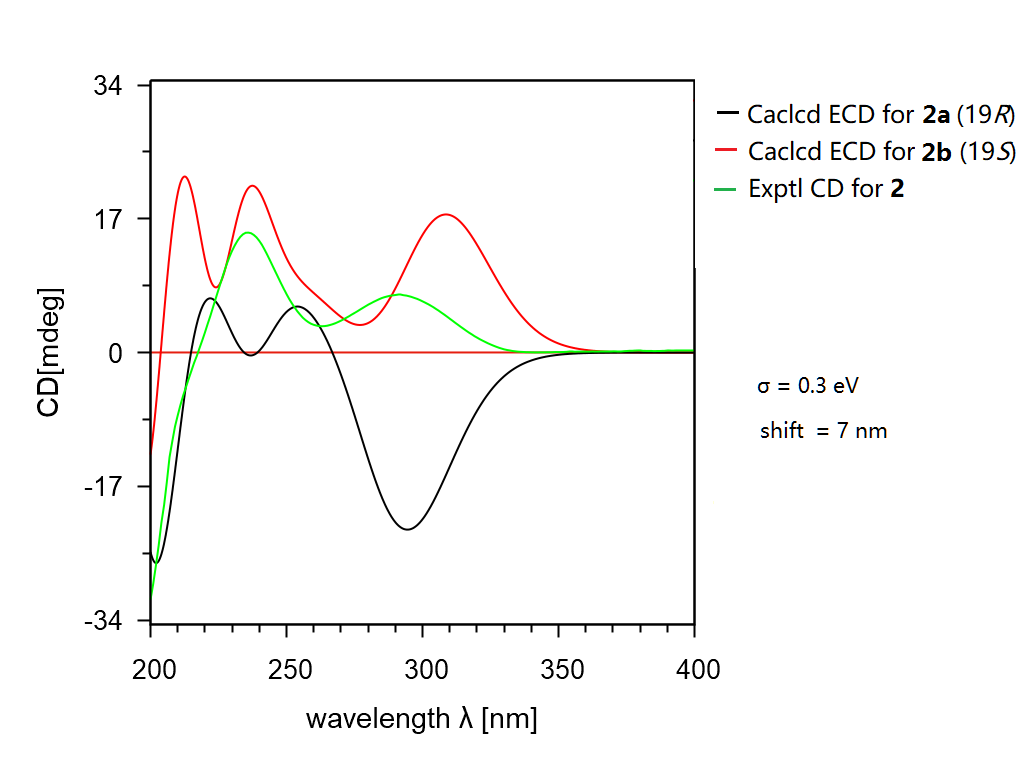


**Figure S65.** Comparison of the calculated ECD spectra for **2a** and **2b** with the experimental spectrum of **2** in gas.

**2. The absolute configuration of C-19 in 3 was determined by calculation of the electronic circular dichroism (ECD).**


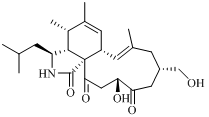

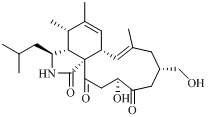


(3*S**,4*R**,5*S**,8*S**,9*S**,16*S**,19*R*)-**3** (**3a**) (3*S**,4*R**,5*S**,8*S**,9*S**,16*S**,19*S*)-**3** (**3b**)

Similarity, there were two possible structures of compound **3**, : (3*S**,4*R**,5*S**,8*S**,9*S**,16*S**,19*R*)-**3** (**3a**) and (3*S**,4*R**,5*S**,8*S**,9*S**,16*S**,19*S*)-**3** (**3b**). We also used MMFF94s force field to search the conformations, which gave 11 and 9 conformers with population higher than 1%, respectively (Figures S66 and S67). These conformers were optimized at the B3LYP/6-31G(d,p) and conducted to theoretical calculation of ECD with TDDFT-B3LYP/6-31G(d,p) level in gas. The calculated ECD curves for **3a** and **3b** and weighted ECD were all generated using SpecDis 1.60 with σ = 0.3 eV, and UV shift -2 nm, respectively. As shown in Figure S68, the calculated ECD spectrum of **3a** matched well with the experimental one, demonstrating the absolute configuration of C-19 to be *R*.


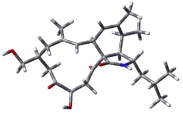

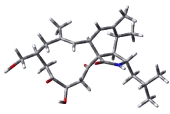

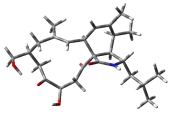


**3a-1 3a-2 3a-3**


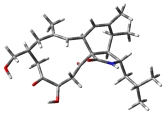

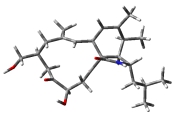

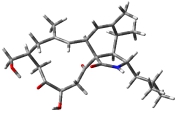


**3a-4 3a-5 3a-6**


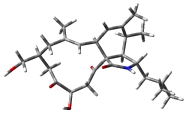

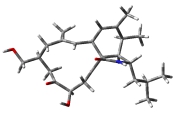

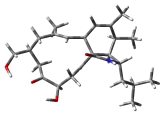


**3a-7 3a-8 3a-9**


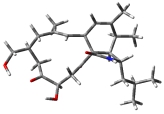

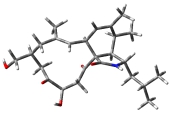


**3a-10 3a-11**

**Figure S66**. Optimized conformers of **3a** (**3a-1-3a-11**).


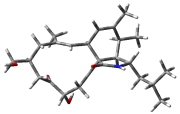


**3b-1 3b-2 3b-3**

**3b-4 3b-5 3b-6**

**3b-7 3b-8 3b-9**

**Figure S67**. Optimized conformers of **3b** (**3b-1-3b-9**).

**Figure S68**. Comparison of the calculated ECD spectra for **3a** and **3b** with the experimental spectrum of **3** in gas.

**3. The absolute configuration of C-18 in 4 was determined by calculation of the electronic circular dichroism (ECD).**

(3*S**,4*R**,5*S**,8*S**,9*S**,16*S**,18*R*)-**4** (**4a**) (3*S**,4*R**,5*S**,8*S**,9*S**,16*S**,18*S*)-**4** (**4b**)

As for compound **4**, theoretical calculation of ECD was also utilized. All steps were the same as former, and two possible structures, (3*S**,4*R**,5*S**,8*S**,9*S**,16*S**,18*R*)-**4** (**4a**) and (3*S**,4*R**,5*S**,8*S**,9*S**,16*S**,18*S*)-**4** (**4b**), had 16 and 19 conformers, respectively. These structures remained 12 and 11 conformers with a 3 kcal/mol energy, respectively (Figures S69 and S70). The calculated ECD curves for **4a** and **4b** and weighted ECD were all generated using SpecDis 1.60 with σ = 0.3 eV, and UV shift -30 nm, respectively. As shown in Figure S71, calculated ECD spectrum of **4a** matched well with the experimental one, which showed the absolute configuration of C-18 in **4** to *R*.

**4a-1 4a-2 4a-3**

**4a-4 4a-5 4a-6**

**4a-7 4a-8 4a-9**

**4a-10 4a-11 4a-12**

**Figure S69**. Optimized conformers of **4a** (**4a-1-4a-12**).

**4b-1 4b-2 4b-3**

**4b-4 4b-5 4b-6**

**4b-7 4b-8 4b-9**

**4b-10 4b-11**

**Figure S70**. Optimized conformers of **4b** (**4b-1-4b-11**).

**Figure S71**. Comparison of the calculated ECD spectra for **4a** and **4b** with the experimental spectrum of **4** in gas.
